# The relative role of plasticity and demographic history in *Capsella bursa-pastoris*: a common garden experiment in Asia and Europe

**DOI:** 10.1093/aobpla/plac011

**Published:** 2022-04-02

**Authors:** Amandine Cornille, Mathieu Tiret, Adriana Salcedo, Huirun R Huang, Marion Orsucci, Pascal Milesi, Dmytro Kryvokhyzha, Karl Holm, Xue-Jun Ge, John R Stinchcombe, Sylvain Glémin, Stephen I Wright, Martin Lascoux

**Affiliations:** Department of Ecology and Genetics, Evolutionary Biology Centre, Uppsala University, 75236 Uppsala, Sweden; Université Paris Saclay, INRAE, CNRS, AgroParisTech, GQE - Le Moulon, 91190 Gif-sur-Yvette, France; Department of Ecology and Genetics, Evolutionary Biology Centre, Uppsala University, 75236 Uppsala, Sweden; Department of Ecology and Evolutionary Biology, University of Toronto, M5S 3B2 Toronto, ON, Canada; Key Laboratory of Plant Resources Conservation and Sustainable Utilization, South China Botanical Garden, Chinese Academy of Sciences, Guangzhou 510650, China; Center of Conservation Biology, Core Botanical Gardens, Chinese Academy of Sciences, Guangzhou 510650, China; Department of Plant Biology, Swedish University of Agricultural Sciences, 750 07 Uppsala, Sweden; Department of Ecology and Genetics, Evolutionary Biology Centre, Uppsala University, 75236 Uppsala, Sweden; Science for Life Laboratory, 752 37 Uppsala, Sweden; Department of Ecology and Genetics, Evolutionary Biology Centre, Uppsala University, 75236 Uppsala, Sweden; Department of Ecology and Genetics, Evolutionary Biology Centre, Uppsala University, 75236 Uppsala, Sweden; Key Laboratory of Plant Resources Conservation and Sustainable Utilization, South China Botanical Garden, Chinese Academy of Sciences, Guangzhou 510650, China; Center of Conservation Biology, Core Botanical Gardens, Chinese Academy of Sciences, Guangzhou 510650, China; Department of Ecology and Evolutionary Biology, University of Toronto, M5S 3B2 Toronto, ON, Canada; Department of Ecology and Genetics, Evolutionary Biology Centre, Uppsala University, 75236 Uppsala, Sweden; UMR CNRS 6553 ECOBIO, Université de Rennes I, 35042 Rennes Cedex, France; Department of Ecology and Evolutionary Biology, University of Toronto, M5S 3B2 Toronto, ON, Canada; Department of Ecology and Genetics, Evolutionary Biology Centre, Uppsala University, 75236 Uppsala, Sweden; Science for Life Laboratory, 752 37 Uppsala, Sweden

**Keywords:** *Capsella bursa-pastoris*, common garden, demographic history, environmental distance, fitness components, phenotypic plasticity

## Abstract

The colonization success of a species depends on the interplay between its phenotypic plasticity, adaptive potential and demographic history. Assessing their relative contributions during the different phases of a species range expansion is challenging, and requires large-scale experiments. Here, we investigated the relative contributions of plasticity, performance and demographic history to the worldwide expansion of the shepherd’s purse, *Capsella bursa-pastoris*. We installed two large common gardens of the shepherd’s purse, a young, self-fertilizing, allopolyploid weed with a worldwide distribution. One common garden was located in Europe, the other in Asia. We used accessions from three distinct genetic clusters (Middle East, Europe and Asia) that reflect the demographic history of the species. Several life-history traits were measured. To explain the phenotypic variation between and within genetic clusters, we analysed the effects of (i) the genetic clusters, (ii) the phenotypic plasticity and its association to fitness and (iii) the distance in terms of bioclimatic variables between the sampling site of an accession and the common garden, i.e. the environmental distance. Our experiment showed that (i) the performance of *C. bursa-pastoris* is closely related to its high phenotypic plasticity; (ii) within a common garden, genetic cluster was a main determinant of phenotypic differences; and (iii) at the scale of the experiment, the effect of environmental distance to the common garden could not be distinguished from that of genetic clusters. Phenotypic plasticity and demographic history both play important role at different stages of range expansion. The success of the worldwide expansion of *C. bursa-pastoris* was undoubtedly influenced by its strong phenotypic plasticity.

## Teaser

The respective role of demography, plasticity and adaptation in the colonization success of plant species remains an intense topic of investigation in evolutionary ecology and genomics. A screening of phenotypic traits of hundreds of genotypes in large-scale common garden experiments in Eastern Asia and Europe shows that both demography and a high phenotypic plasticity underlie the success of the tetraploid and self-fertilizing species, *Capsella bursa-pastoris*, the shepherd’s purse, at different stages of expansion. This study provides insight into the causes of the ecological success of a plant species during range expansion.

## Introduction

Range expansion can leave a strong footprint on current patterns of genetic and phenotypic variation (Excoffier and Ray 2008; Excoffier *et al.* 2009). The factors influencing the speed of range expansion and range limits, and their impacts on genetic and phenotypic variation are still being debated (Angert *et al.* 2020). Current and future threats to biodiversity, including invasive species outbreaks and their consequences for ecosystem health and services, have made this question more relevant than ever.

The speed of range expansion of sessile organisms generally depends on reproductive capacity, seed dispersal efficiency and the species’ ability to establish itself successfully in new environments (Sheth *et al.* 2020). Establishment success may depend on phenotypic plasticity in the short term, and on adaptive capacity, in the long term (Lande 2009; Hendry 2016), especially if the new environment and that of the parents differ in terms of abiotic (e.g. temperature, light, photoperiod, soil composition) and biotic conditions (e.g. neighbouring plants, local microbiomes, pathogens, pollinator communities). Phenotypic plasticity is defined here as the ability of a given genotype to produce different phenotypes in different environments (Bradshaw 1965; Grenier *et al.* 2016 and references therein), and genetic adaptation as evolution through natural selection whereby the average fitness of a population gradually increases in a given environment (Linhart and Grant 1996; Chevin *et al.* 2010 and references therein). A successful expansion will correspond to a particular combination of these two evolutionary mechanisms, which are not mutually exclusive (Hendry 2016). Species with a worldwide distribution inevitably face a large array of environments and signatures of both phenotypic plasticity and adaptation are likely to be observed (Lande 2015). Investigating plasticity in newly established populations is fraught with difficulties, since the age of populations is generally hard to estimate and patterns of phenotypic plasticity may vary depending on the time since colonization. Indeed, theoretical models indicate that while adaptation to a new extreme environment may lead to a transient increase in phenotypic plasticity, this can be followed by a second period of genetic assimilation, which, perhaps unexpectedly, is associated with a decrease in plasticity (Lande 2015). These complex dynamics could explain why different studies on phenotypic plasticity of colonizing species led to divergent conclusions: during the colonization of new environments, high mean phenotypic plasticity has been under- (e.g. Daehler 2003) or over-represented (e.g. Davidson *et al.* 2011; Godoy *et al.* 2011). Likewise, while evolutionary forces underlying adaptation in populations at equilibrium are fairly well documented, the literature is more limited for recent and marginal populations that are usually characterized by non-equilibrium demographics. In such populations, random genetic drift can play a larger part and obscure the effect of adaptive forces, making the patterns of genetic variation harder to interpret (Excoffier *et al.* 2009; Gilbert *et al.* 2017), as illustrated in *Mercurialis annua* (González-Martínez *et al.* 2017) or in *Arabidopsis lyrata* (Willi *et al.* 2018). In summary, as illustrated in the review of Hendry (2016), estimating the relative role of phenotypic plasticity and past adaptation during colonization remains a very open question.

Phenotypic plasticity and demographic history, and their interplay, have probably played a key role in the worldwide success of shepherd’s purse, *Capsella bursa-pastoris* (Brassicaceae). The shepherd’s purse is a self-fertilizing colonizer of recent allopolyploid origin. The shepherd’s purse arose some 100 000 years ago from hybridization between the diploid self-fertilizing *Capsella orientalis* and the outcrossing *Capsella grandiflora* (Douglas *et al.* 2015). Autogamy and allopolyploidy could partly explain why *C. bursa-pastoris* has an almost worldwide distribution while its two parents are restricted to specific areas: from Central Asia to eastern Europe for *C. orientalis*, and only the mountains of north-west Greece and Albania for *C. grandiflora* (Hurka *et al.* 2012). The shepherd’s purse is genetically structured in three distinct clusters: eastern Asia (ASI), Middle East and northern Africa (ME) and Europe and the Russian Far East (EUR) (Cornille *et al.* 2016). Wesse *et al.* (2021) found a similar clustering, where the Middle Eastern and northern African cluster corresponds to their Mediterranean lineage and the European and the Russian Far East to their temperate lineage. Demographic inferences showed that the three clusters (ASI, EUR, ME) resulted from a range expansion that started either from the Middle East or Europe (the starting point is not known with certainty), and was followed by a subsequent colonization event towards Asia. This recent worldwide spread was, in some cases, likely associated with human migrations, as for instance the spread to eastern Siberia of western European accessions (Cornille *et al.* 2016), or of southern European and Middle Eastern accessions to North America (Hurka and Neuffer 1997; Cornille *et al.* 2016). The shift from outcrossing to self-fertilization (a.k.a. selfing) confers ‘reproductive assurance’, which is expected to facilitate colonization of new environments as only one or a few individuals are required to establish a new population (Baker *et al.* 1965; Pannell *et al.* 2015). The benefit of self-fertilization might be short-lived, however, as the lack of genetic diversity and effective recombination is expected to limit adaptation and to lead to a genome-wide accumulation of deleterious mutations (a.k.a. genetic load; Heller and Smith 1978; Hollister *et al.* 2015; Glémin *et al.* 2019). The Asian cluster of *C. bursa-pastoris* shows such a genetic load (Kryvokhyzha *et al.* 2019a). In agreement with these predictions, selfing species tend to have larger ecological ranges (Grossenbacher *et al.* 2015) but decreasing niche breadth over time (Park *et al.* 2018) compared to their outcrossing congeners, aligned with the ‘evolutionary dead end’ hypothesis (Stebbins 1957; Takebayashi and Morrell 2001 and references therein). The phenomenon of a decreased niche breadth is even more pronounced during a colonization process (e.g. Slatkin and Excoffier 2012; González-Martínez *et al.* 2017).

**Figure 1.**
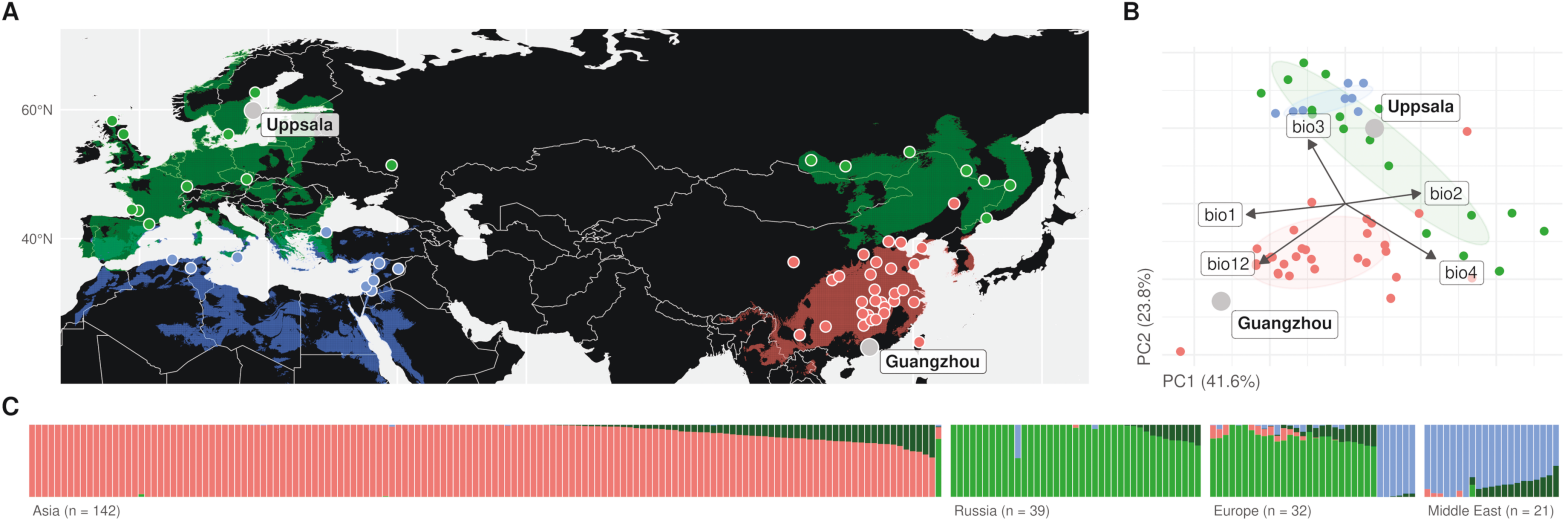
Experimental design and associated ecology and population genetic structure of samples of *Capsella bursa-pastoris*. (A) Geographical distribution of the common gardens (labelled grey dots) and the 59 sampling sites (small dots). The colour of each sampling site matches its genetic cluster (panel C): red for the Asian cluster (ASI), green for the European cluster (EUR) and blue for the Middle Eastern cluster (ME). WorldClim database was used to construct climatic ranges based on similarity to sampling sites (i.e. within a 60 % confidence ellipse on the PCA, panel B); climatic ranges are coloured according to genetic clusters. (B) Principal component analysis of the 19 bioclimatic variables; only the most significant bioclimatic variables are displayed: the *annual mean temperature* (bio1), the *mean diurnal range* (bio2), *isothermality* (bio3), *temperature seasonality* (bio4) and the *annual precipitation* (bio12). Common gardens (grey dots) and sampling sites (small dots) are grouped in three geographical regions (with 60 % confidence ellipses): Asia (red), Europe (green) and the Middle East (blue). The first and the second components (PC1 and PC2), respectively, captured 41.6 % and 23.8 % of the climatic variance. (C) Co-ancestry coefficients inferred with ADMIXTURE (*K* = 4). The value of *K* was chosen as the inflection point on the cross-validation. Three clusters out of four matched the geographical regions: Asia (142 accessions), Europe + Russia (32 + 39 accessions) and the Middle East (21 accessions). The fourth cluster was shared among populations. The rightmost accession of Asia is from Harbin (close to the Russian borders), and the rightmost accessions of Europe were from South Italy. For colour figure refer online version.

Hence, understanding the causes of ecological success of a species requires the estimation of the respective roles of phenotypic plasticity and demographic history during its range expansion, and *C. bursa-pastoris* is a perfect model species for this. To that end, we implemented an experiment with two large common gardens located in two contrasting environments (i.e. environments with differing day length, temperature, moisture, soil and plant community). First, to capture the diversity of environmental conditions that *C. bursa-pastoris* faced during its range expansion, we installed two common gardens at extreme latitudes in Eurasia, one in East Asia and one in Northern Europe (Fig. 1A). Second, to capture the demographic history of *C. bursa-pastoris,* we used a comprehensive sample of populations from Europe, Asia, North Africa and the Middle East (Fig. 1B and C). Third, to be able to characterize phenotypic variation and individual performances, we measured several life-history and phenological traits, some of which are main fitness components. This experimental set-up provided us with a solid framework to assess the effects of (i) the environment through a large common garden experiment, (ii) the genetic clusters associated to past demographic history and (iii) the distance in terms of bioclimatic variables between sampling sites and common garden, i.e. the environmental distance. Finally, we discussed the relative importance of phenotypic plasticity and demographic history in the light of *C. bursa-pastoris* colonization history and its expansion load.

## Materials and Methods

### Plant materials

#### Sampling.

We used a collection of 232 accessions—offspring of a single self-fertilizing mother plant—from 59 sites distributed across Europe, Asia, North Africa and the Middle East (Fig. 1A; **see****Supporting Information—Fig. S1**). These accessions were previously described in Cornille *et al.* (2016) (see also Kryvokhyzha *et al.* 2016; Kryvokhyzha *et al.* 2019b). The collection was constituted to represent the global population structure of *C. bursa-pastoris* which is divided in at least three main genetic clusters (Cornille *et al.* 2016): the European (EUR), Middle Eastern (ME) and Asian (ASI). Sixty-six accessions come from EUR, 27 from ME and 139 from ASI **[see****Supporting Information****]**. Each accession was represented by four to six progenies in each common garden, so that the study included in total 2403 plants, 1312 in Uppsala (767 from ASI, 389 from EUR and 156 from ME) and 1091 in Guangzhou (555 from ASI, 374 from EUR and 162 from ASI).

#### Phenotypes.

After senescence, we recorded for each plant several morphological traits: the *final height of the highest inflorescence* (in cm), the *number of basal inflorescences* and the *number of fruits along a section of 10 cm* in the middle of the main inflorescence. To estimate the overall performance of each individual, and as a proxy for fitness, we calculated the total number of fruits per individual as the product of the aforementioned three traits.

We also measured four phenological traits: *germination time*, *bolting time* (i.e. time until differentiation of the bud from vegetative parts indicating the initiation of the reproductive period), *flowering time* (i.e. time until the appearance of the first opened flower) and *senescence time* (i.e. time until the drying state of the plant). In addition, we calculated the four inter-event periods: time between *sowing* and *germination* (*GP*), time between *germination* and *bolting* (*BP*), time between *bolting* and *flowering* (*FP*) and time between *flowering* and *senescence* (*SP*), also called *flowering duration.* At *flowering time*, we recorded two additional morphological traits: the *number of rosette leaves* and the *maximum diameter of the rosette* (in cm).

### Common gardens

#### Localization.

One common garden was located in Uppsala (59°51ʹN, 17°37ʹE, Sweden), and the second one in Guangzhou (23°11ʹN, 113°21ʹE, China). Experiments were conducted at the natural growing period of *C*. *bursa-pastoris* in each common garden: for 139 days from May to September 2014 in Uppsala, and for 193 days from November 2014 to May 2015 in Guangzhou. Throughout the experimental period, day length becomes longer in Uppsala, and shorter in Guangzhou.

#### Environmental data.

Environmental conditions were monitored daily at ground level using temperature and humidity sensors (TGP-4017®, Gemini Data Loggers; Chichester, West Sussex, UK). The overall environmental conditions were reported in **Supporting Information—Fig. S2****and****Tables S1****and****S2**. In Uppsala, climatic conditions were rather cold and wet (15.2 ± 5.1 °C, 76.8 ± 14.1 %), while in Guangzhou accessions experienced warm and humid weather (19.3 ± 5.0 °C, 81.1 ± 11.4 %).

Day length was obtained with the package *geosphere* (function *daylength*; Hijmans *et al.* 2015) in the R environment for statistical computing (R-3.6.3; R Core Team 2020). The photoperiod was longer in Uppsala (16.9 ± 1.7 h, ranging from 13 to 18.8 h) than in Guangzhou (11.9 ± 1.0 h, ranging from 10.7 to 13.5 h). Climate data were downloaded from WorldClim database (19 bioclimatic variables, 2.5 arc-minute resolution, from 1960 to 2000; Fick *et al.* 2017).

### Experiment protocol

#### Seed preparation.

In both common gardens, to reduce maternal effects, seeds collected from the field were first sown and grown under controlled conditions in growth chambers (55 % moisture, 22 °C, 12h:12h light:darkness cycles). Their progenies were then used to establish the common gardens. More specifically, about 20 seeds per accession were surface-sterilized the same day and germinated in Petri dishes (one per accession), with Murashige and Skoog (MS) medium and agar (see protocol in Kryvokhyzha *et al.* 2016). Petri dishes were then all stratified for 7 days at 4 °C in the dark to promote germination (avoiding differential dormancy release; see Salisbury 1963; Neuffer and Hurka 1988), and then placed in a greenhouse with no additional light or heating, in order to protect seeds from rainfall and to facilitate acclimation to outdoor conditions. Petri dishes were randomized over tables and moved every day to avoid micro-environmental effects and were left in the greenhouse until seedlings reached a four-leaf stage.

#### Transplantation.

In both common gardens, once seedlings reached a four-leaf stage, they were thinned to one per pot (7 cm × 7 cm) containing standard plant nursery soil mixed with water (at Uppsala: yrkesplantjord, Wexthuset, Enhörna, Sweden; at Guangzhou, Jiffy substrates; Zwijndrecht, The Netherlands). The pots were randomly placed in the greenhouse, and left 7 days. During the 7 days, the pots were watered automatically twice a week, and randomly moved every day to avoid the impact of micro-environmental variation in the greenhouse. After 7 days, the pots were pierced on the bottom and placed outside in the common garden, so that the plants were using the soil from Uppsala or Guangzhou regardless of the initial nursery soil. The pots were dispatched into six blocks (1 m × 3.2 m, grids of 9 × 30) arranged at 2 m spacing and containing standard soil. Each block contained one replicate per accession, with a total of six replicates per accession. When less than six seedlings from an accession germinated, seedlings from another accession of the same sampling site complemented the block, in order to keep a uniform individual density. Apart from the initial shading and watering, no additional support was provided to the seedlings. The experiment lasted until the senescence of the last plant, i.e. when the last plant dried up but had not yet shed its fruits.

### Statistical analyses

#### Effect of the genetic cluster.

The common gardens were first analysed with the following mixed model:


$$\begin{array}{l} Y_{ijklm}∼\mu +c_{i}+\;s_{j}+\;B_{k}+\;A_{l}+\;e_{ijklm} \end{array}$$ (1)

where ‘*Y*_*ijklm*_’ is a phenotype of the individual *m* from the accession *l* of the genetic cluster *i* in the block *k* of the common garden *j*, ‘*μ*’ is the overall mean, ‘*c*_*i*_’ is the fixed effect of the *i*th genetic cluster, *s*_*j*_ is the fixed effect of the *j*th common garden (Uppsala or Guangzhou), ‘*B*_*k*_’ and ‘*A*_*l*_’ are the uncorrelated random effects of the *k*th blocks and the *l*th accession, respectively (the accession’s effect is nested in the cluster’s effect) and ‘*e*_*ijklm*_’ is the residual. The residual was fitted to different distributions according to the lowest Akaike information criterion and depending on the trait (Table 1). Model fitting relied on the R package *lme4* (function *lmer* and *glmer.nb*; Bates *et al.* 2015). In order to account for the difference in sample sizes of the genetic clusters, additional analyses were also carried out after downsampling. Unless otherwise specified, the results can be assumed to be robust to uneven sample size (for further details, **see****Supporting Information**). To remove the unbalance in block design that was caused by experimental issues (e.g. failed germinations, climatic events), the block design was balanced before each statistical analysis by downsampling the data set down to four repetitions per accession per common garden **[see****Supporting Information****]**. Additional analyses were run within common garden to focus on the effect of genetic clusters.

**Table 1.** Effects of the genetic cluster (i.e. Middle East, Asia or Europe) on each trait in each common garden (Uppsala or Guangzhou), with an analysis of variance of model (1), in *Capsella bursa-pastoris*. Mean and standard deviation (SD), statistics (*χ*^2^) and df of the type II Wald chi-square test. GP: time between *sowing* and *germination*; BP: time between *germination* and *bolting*; FP: time between *bolting* and *flowering*; SP: time between *flowering* and *senescence*. The distributions of the residual are: negative binomial (NB), normal (N) or normal with the starting date as an additional variable (N*). Significant values are bolded. Significance levels are: ***P < 0.001; **P < 0.01; *P < 0.05; ^n.s.^*P* > 0.05.

Trait	Genetic cluster effect						Residual
	Uppsala			Guangzhou			
	Mean ± SD	*χ* ^2^	df	Mean ± SD	*χ* ^2^	df	
No. of fruits	224 ± 174	7.16*	2	1069 ± 808	40.61***	2	NB
Height (in cm)	23.3 ± 8.6	78.33***	2	60.1 ± 19.0	36.21***	2	N
No. of fruits over 10 cm	31.0 ± 14.1	9.46**	2	37.3 ± 13.9	17.5***	2	N
No. of primary branches	2.91 ± 1.47	0.64^n.s.^	2	4.12 ± 1.91	11.11**	2	N
No. of secondary inflorescences	5.78 ± 5.00	18.80***	2	3.87 ± 1.82	1.14^n.s.^	2	NB
No. of rosette leaves	20.7 ± 10.3	136.40***	2	21.9 ± 10.8	44.31***	2	N
Rosette diameter (in cm)	10.6 ± 4.3	100.22***	2	18.9 ± 8.9	24.15***	2	N
Germination (in days)	3.94 ± 3.55	43.80***	2	1.23 ± 1.32	138.83***	2	N
Bolting (in days)	30.6 ± 5.7	26.58***	2	61.7 ± 12.1	8.93*	2	N
BP (in days)	27.0 ± 5.5	101.79***	2	60.4 ± 12.2	145.14***	2	N*
Flowering (in days)	35.8 ± 5.5	23.32***	2	72.0 ± 12.5	17.94***	2	N
FP (in days)	7.39 ± 3.1	1.36^n.s.^	2	10.4 ± 3.0	20.18***	2	N*
Senescence (in days)	84.0 ± 6.4	10.14**	2	139 ± 11	8.72*	2	N
SP (in days)	48.8 ± 8.3	21.33***	2	65.6 ± 13.6	11.29**	2	N*

For the four inter-event phenological periods, the starting date of each time span was included in the model as an additional fixed effect. Statistical significance of the genetic cluster effect was assessed with a type II Wald chi-square test, and the difference between genetic clusters was assessed with Tukey’s HSD (honestly significant difference) tests (for expected values; using the function *glht* of the package *multcomp*; Hothorn *et al.* 2008) and Fisher’s *F*-tests (for variances; using the function *var.test* of the package base *stats*).

#### Effect of environmental distance.

In a worldwide scale experiment, what matters most for phenotypic variation is the environmental differentiation rather than the geographical distance. Following the approach of Lovell *et al.* (2021), we considered the ‘environmental distance’, defined as the distance between a sampling site and the common garden in terms of bioclimatic variables. The distance, reported in **Supporting Information—Table S3**, was computed on the principal component analysis (PCA) of the 19 bioclimatic variables, truncated to the first 10 principal components, explaining 94.21 % of the total variance (Fig. 1B; see also an alternative in Samis *et al.* 2019). To investigate the effect of environmental distance, the following linear mixed model was fitted to the data:


$$\begin{array}{l} Y_{ijkl}∼\;\mu \;+\;d_{k}+\;c_{i}+\;B_{j}+\;A_{k}+\;e_{ijkl} \end{array}$$ (2)

where ‘*d*_*k*_’ is the fixed effect of the environmental distance of the *k*th accession between its sampling site and the common garden, and the other terms are as described above for model (1). An additional statistical model was introduced to investigate the sole effect of environmental distance, i.e. excluding the genetic cluster effect, and was formulated as follows:


$$\begin{array}{l} Y_{ijkl}∼\;\mu \;+\;d_{k}+\;B_{j}+\;A_{k}+e_{ijkl} \end{array}$$ (3)

where the terms are as described above for models (1) and (2). Statistical significance of *d*_*k*_ in models (2) and (3) was assessed using an analysis of deviance (type II Wald chi-square test). Additional analyses were run within a genetic cluster to focus on the effect of environments.

Partial leverage values in models (2) and (3) were particularly high in Guangzhou for accessions sampled in north-eastern China (Harbin) and north-western China (Xining; **see****Supporting Information—Fig. S3**), despite the robustness of the genetic clustering (Cornille *et al.* 2016; Fig. 1C; **see****Supporting Information**). To remove an artificially large environmental distance despite the genetic proximity, these populations were excluded from models (2) and (3), excluding 93 plants across common gardens (9 accessions), and leaving 2310 plants in total (223 accessions).

#### Measure of phenotypic plasticity.

In order to quantify phenotypic plasticity, we modelled each accession as follows:


$$\begin{array}{l} Y_{ijkl}∼\;s_{j}+\;e_{ijkl} \end{array}$$ (4)

where the terms are as described above in model (1). For each accession, we estimated the site effect of Guangzhou and the site effect of Uppsala, and studied the plasticity through the difference between these estimated site effects. In order to study the magnitude of plasticity—not its direction—we considered the absolute value, hereafter denoted *P*-score. Statistical significance of the *P-*score was assessed with a Student’s *t*-test.

When assessing the association between the *P-*score and a phenotype, we used the following model:


$$\begin{array}{l} Y_{ijkl}∼\;\mu \;+\;P_{l}+\;e_{ijkl} \end{array}$$ (5)

where *P*_*l*_ is the *P*-score of the *l*th accession estimated in model (4), and the other terms are as defined in model (1). We studied model (5) under different conditions: considering phenotypes across common gardens, within Uppsala, or within Guangzhou. Statistical significance of the *P*-score in model (5) was assessed with a Fisher’s *F*-tests.

## Results

### Between common gardens, environmental variation was the main explanatory factor of phenotypic variation

**Figure 2.**
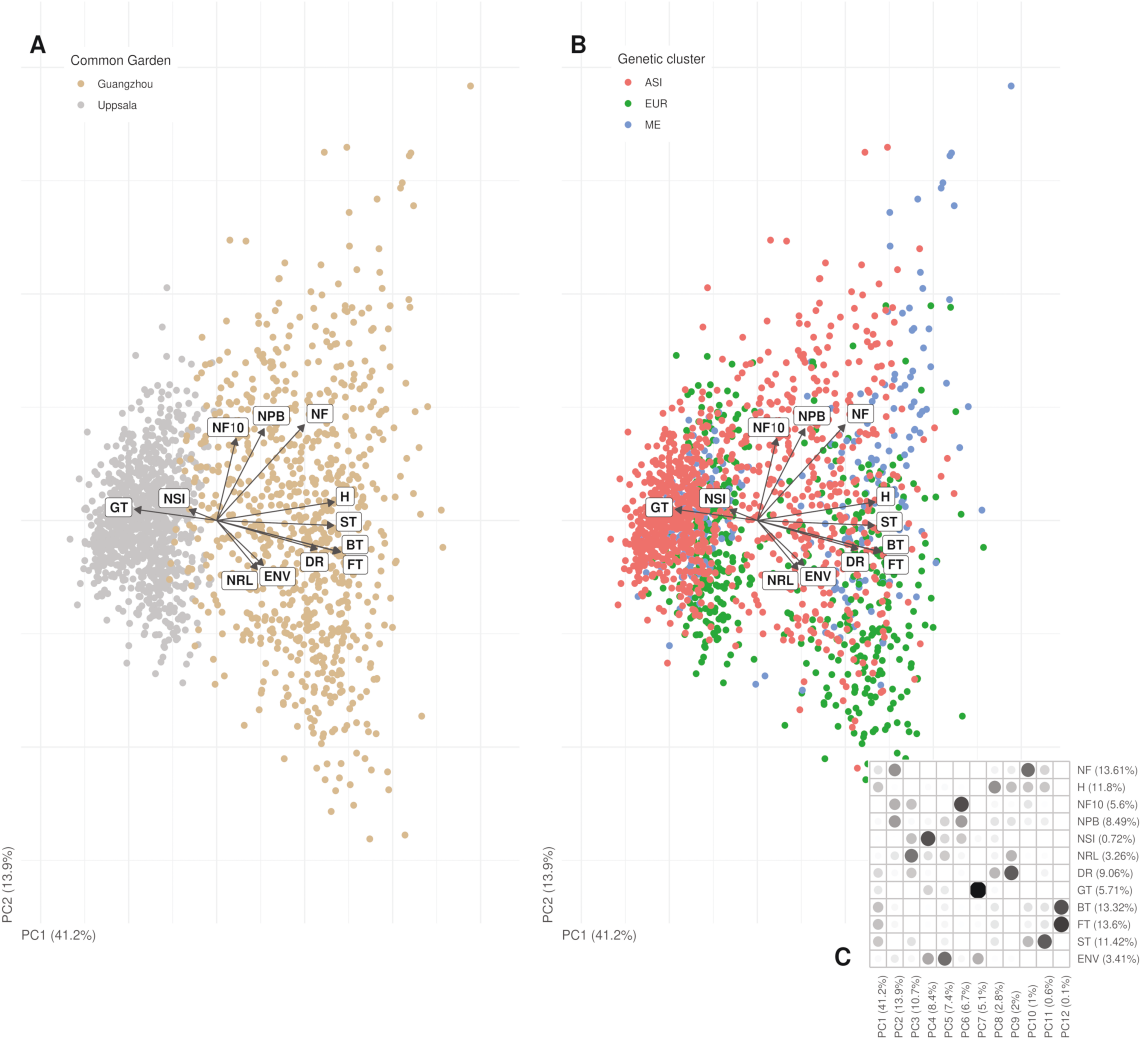
Phenotypic variation of *Capsella bursa-pastoris*. Principal component analysis based on the following phenotypic traits: *number of fruits* (NF), *height of the main inflorescence* (H), *number of fruits over 10 cm* (NF10), *number of primary branches* (NPB), *number of rosette leaves* (NRL), *diameter of rosette* (DR), *germination time* (GT), *bolting time* (BT), *flowering time* (FT), *senescence time* (ST) and *environmental distance* (ENV). The first and the second components (PC1 and PC2), respectively, captured 44.1 % and 14.7 % of the phenotypic variance. (A) The colours match the common gardens: grey for Uppsala and beige for Guangzhou. (B) The colours match the genetic clusters: red for ASI, green for EUR and blue for ME. (C) Correlation between variables and principal components, along with their contributions (in % of the total variance). Large and dark circles correspond to high correlations. For colour figure refer online version.

The phenotypes of *C. bursa-pastoris* are mostly explained by the environments **[see****Supporting Information—Fig. S4****]**, as the site effect in model (1) was significant for all phenotypes (apart from the *number of rosette* leaves and *FP*) at an adjusted Bonferroni cut-off of 0.0036 (all *χ*^2^ > 14.0, degree of freedom or df = 1, *P* < 0.001). The PCA of the phenotypes also supported this trend (Fig. 2A), as the location—Uppsala or Guangzhou—was strongly correlated with the first component PC1 (explaining 41.2 % of the variance). Accordingly, the measure of phenotypic plasticity—the *P-*score—was significant at the same adjusted Bonferroni cut-off for every phenotype (all *t* > 3.79, df_1_ = 1, df_2_ = 39–67, *P* < 0.0013). The environment was much more decisive than genetic differentiation in explaining phenotypic differences, as the genetic cluster effect explained significantly less than the site effect in model (1) for most of the traits **[see****Supporting Information—Table S4****]**, and as the PCA revealed the genetic differentiation only from the third component **[see****Supporting Information—Fig. S5****]**.

As a consequence of this strong environmental effect, accessions were markedly different in the two common gardens. In Uppsala, accessions performed poorly for most morphological phenotypes: the performance was lower (Table 1) for traits positively correlated to the number of fruits—a proxy of fitness—such as *height* (Pearson *ρ* = 0.63, *P* < 0.001), *number of fruits over 10 cm* (*ρ* = 0.65, *P* < 0.001) and *number of primary branches* (*ρ* = 0.66, *P* < 0.001). Similarly, the phenology which is much less correlated to the number of fruits (|*ρ*| < 0.16, *p* < 0.001) shifted significantly between Uppsala and Guangzhou **[see****Supporting Information—Fig. S6****]**, with *bolting time, flowering time* and *senescence time* being longer in Guangzhou (respectively, 61.7 ± 12.1 days, 72.0 ± 12.5 days and 139 ± 11 days) than in Uppsala (30.6 ± 5.7 days, 35.8 ± 5.7 days and 84.0 ± 6.4 days). Since *C. bursa-pastoris* is a facultative long-day species (i.e. flowering is inhibited by short day length; Hurka *et al.* 1976), flowering was, as expected, inhibited during a longer period in Guangzhou than in Uppsala (Ceplitis *et al.* 2005).

**Figure 3.**
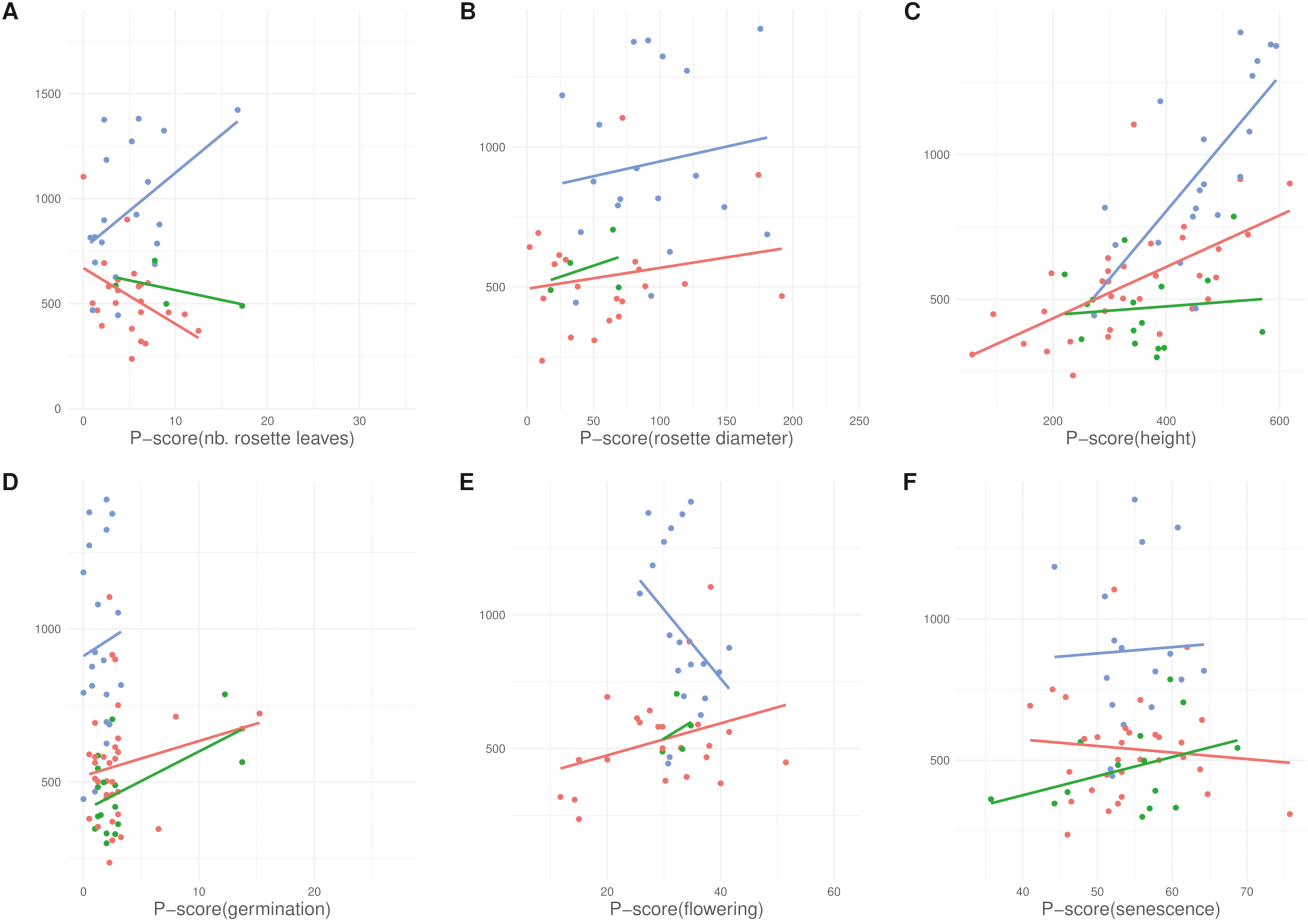
Phenotypic plasticity of *Capsella bursa-pastoris*. The mean number of fruits across common gardens (*y*-axis) as a function of the *P*-score (*x*-axis). Each dot represents one accession. Genetic clusters: ASI: red; EUR: green; ME: blue. (A) Number of rosette leaves. (B) Rosette diameter (in cm). (C) Height (in cm). (D) Germination time (in days). (E) Flowering time (in days). (F) Senescence time (in days). For colour figure refer online version.

Accessions with the highest plasticity in some morphological traits were the most successful in terms of number of fruits—a proxy of fitness, since the *P*-scores of *height*, *number of fruits over 10 cm* and *number of primary branches* were significantly associated with the number of fruits in model (5) (*F* > 17.81, df_1_ = 1, df_2_ = 67, *P* < 0.001; **see****Supporting Information—Table S5**). The significant associations were due to the most plastic accessions having more fruits in Guangzhou but not in Uppsala: *P-*scores in model (5) were significant at the adjusted Bonferroni cut-off of 0.0036 in Guangzhou for *height*, *number of fruits over 10 cm* and *number of primary branches* (*F* > 22.24, df_1_ = 1, df_2_ = 67, *P <* 0.001), but significant for none of the phenotypes in Uppsala (*F* < 8.20, df_1_ = 1, df_2_ = 39–67, *P* > 0.006). In Uppsala, compared to Guangzhou, the most plastic accessions in terms of number of fruits had smaller *height* (model (5), *P*_*l*_ = −0.02, *P* = 0.67), *number of fruits over 10 cm* (model (5), *P*_*l*_ = −0.44, *P* < 0.001) and *number of primary branches* (model (5), *P*_*l*_ = −0.22, *P* = 0.007). In other words, plasticity in some morphological traits was beneficial in terms of number of fruits only in Guangzhou. In Uppsala, plasticity only affected morphology, but not the number of fruits. None of the associations between the number of fruits and the *P*-scores were significantly specific to a genetic cluster (Fig. 3), apart from *P*-score of *height* in ASI and ME (*F* > 19.28, df_1_ = 1, df_2_ = 18–31, *P <* 0.001; **see****Supporting Information—Table S5**).

### Within each common garden, most of the phenotypic variation is explained by the genetic clusters

**Figure 4.**
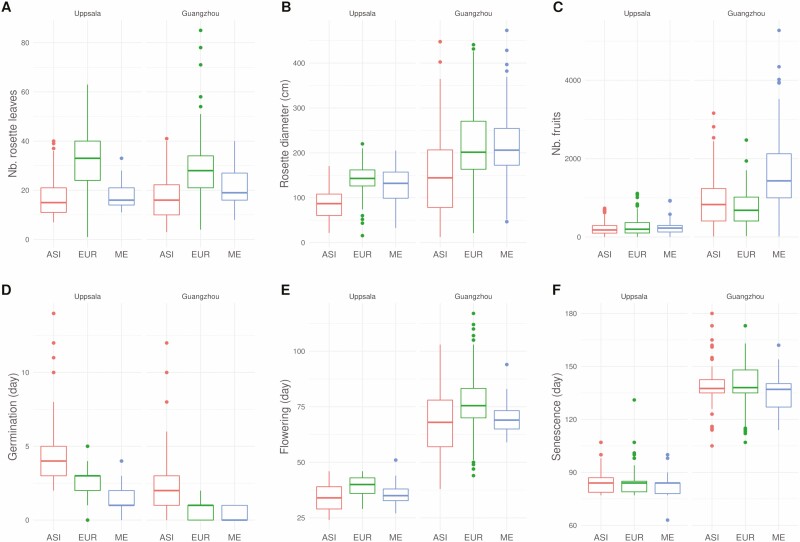
Phenotypic variation among genetic clusters and common gardens for *Capsella bursa-pastoris*. Box plots of the most significant phenotypes in *C. bursa-pastoris*, in each common garden, for each cluster (ASI: red; EUR: green; ME: blue). For each trait and each common garden, block design was balanced as described in **Supporting Information**. (A) Number of rosette leaves. (B) Rosette diameter (in cm). (C) Total number of fruits. (D) Germination time (in days). (E) Flowering time (in days). (F) Senescence time (in days). For colour figure refer online version.

Within a common garden, each accession experienced the same environment, so that only genetics and the interaction between genetics and environments (G x E) can explain the variation among accessions. Statistical analyses of model (1) within each common garden suggested a genetic origin of the phenotypic variation (Table 1) as the genetic cluster effect was, at a Bonferroni adjusted cut-off of 0.0018 (28 comparisons: 14 phenotypes in two common gardens), significant for 9 traits out of 14 in Uppsala (average *χ*^2^ = 41.4, df = 2), and 9 out of 14 in Guangzhou (average *χ*^2^ = 37.6, df = 2). Genetic cluster had a significant effect in both common gardens for most of the traits (*χ*^2^ > 7.16, df = 2, *P* < 0.05; Table 1). For a handful of traits, genetic cluster had a significant effect in only one common garden: not significant in Uppsala for the *number of primary branches* and *FP* (*χ*^2^ = 0.64, df = 2, *P* =0.73), while significant in Guangzhou (*χ*^2^ = 11.11, df = 2, *P* < 0.01); not significant in Guangzhou for the *number of secondary inflorescences* (*χ*^2^ = 1.14, df = 2, *P* = 0.57), while significant in Uppsala (*χ*^2^ = 18.80, df = 2, *P* < 0.001). Across the common gardens, the relation of phenotypes between the genetic clusters remained stable between ASI and EUR, and stable for some traits (*number of rosette leaves*, *germination*, *flowering*) with ME (Fig. 4). For the *number of fruits*, Middle Eastern accessions were significantly higher (Tukey’s HSD > 4.54, *P* < 0.05) than the other clusters in Guangzhou (Table 2), although in Uppsala all three genetic clusters are quite remarkably similar.

**Table 2.** Differences in phenotypes among *Capsella bursa-pastoris* genetic clusters in each common garden (Uppsala and Guangzhou) in model (1). Statistics of the Tukey contrast analysis. Significant values are bolded. Significance levels are: ***P < 0.001; **P < 0.01; *P < 0.05; ^n.s.^*P* > 0.05.

Traits	Uppsala			Guangzhou		
	EUR–ASI	ME–ASI	ME–EUR	EUR–ASI	ME–ASI	ME–EUR
No. of fruits	2.39*	1.76^n.s.^	−0.05^n.s.^	−2.75*	4.54***	6.34***
Height	8.69***	3.70***	−2.47*	2.63*	5.99***	3.53**
No. of fruits over 10 cm	−1.58^n.s.^	2.13^n.s.^	3.07**	−2.83*	2.02^n.s.^	4.10***
No. of primary branches	0.49^n.s.^	−0.49^n.s.^	−0.78^n.s.^	−1.71^n.s.^	2.01^n.s.^	3.33**
No. of secondary inflorescences	3.55**	3.34**	0.64^n.s.^	0.86^n.s.^	0.91^n.s.^	0.17^n.s.^
No. of rosette leaves	11.34***	0.11^n.s.^	−8.44***	6.64***	1.92^n.s.^	−3.30**
Rosette diameter	9.22***	6.06***	−1.74^n.s.^	4.29***	3.74***	0.25^n.s.^
Germination	−4.83***	−5.60***	−1.95^n.s.^	−9.97***	−9.27***	−1.53^n.s.^
Bolting	4.10***	−1.89^n.s.^	−4.63***	2.93**	0.48^n.s.^	−1.73^n.s.^
BP	8.64***	7.21***	0.56^n.s.^	10.32***	9.41***	1.29^n.s.^
Flowering	4.71***	0.12^n.s.^	−3.46**	4.11***	0.51^n.s.^	−2.68*
FP	−0.65^n.s.^	0.80^n.s.^	1.17^n.s.^	3.81**	−0.90^n.s.^	−3.87***
Senescence	−1.24^n.s.^	−3.15**	−2.11^n.s.^	1.06^n.s.^	−2.22^n.s.^	−2.95**
SP	−4.54**	−0.34^n.s.^	3.15**	−3.29**	−0.43^n.s.^	2.00^n.s.^

Genetic cluster effects for rosette characters (*number of rosette leaves* and *maximum rosette diameter*) were much stronger than those of other phenotypes in Uppsala (*χ*^2^ > 100.22, df = 2, *P* < 0.001), though less pronounced in Guangzhou (*χ*^2^ > 24.15, df = 2, *P* < 0.001). Rosettes of European accessions were significantly larger and had more leaves than those of Asian accessions in both common gardens (Tukey’s HSD > 4.29, *P* < 0.001; Table 2), and had significantly more leaves than the Middle Eastern accessions in both common gardens (Tukey’s HSD < −3.30, *P* < 0.01). The Middle Eastern accessions had significantly larger rosettes than Asian accessions in both common gardens (Tukey’s HSD > 3.74, *P* < 0.001).

Phenological variation was also strongly determined by the genetic cluster of the accession in Uppsala (average *χ*^2^ = 32.6, df = 2) and in Guangzhou (average *χ*^2^ = 50.1, df = 2). The Asian cluster exhibited late germination, early bolting, early flowering and late senescence, and therefore a long flowering duration. In contrast, the European cluster germinated early, but bolted, flowered and withered late. European accessions’ late flowering (but not all; see Neuffer 2011) partly explains their larger rosette, since they had more time for vegetative growth. Accessions from the Middle Eastern cluster did not follow any particular trend, showing a certain amount of plasticity in their phenological response. Pairwise ranking of phenological traits was consistent among common gardens (Table 2).

When focusing on the total number of fruits, the European cluster significantly outperformed the Asian cluster in Uppsala (Tukey’s HSD = 2.39, *P* < 0.05), although this trend was no longer significant (*P* = 0.38) after dowmsampling. In Guangzhou the Middle Eastern cluster outperformed both the Asian and the European clusters (Tukey’s HSD > 4.54, *P* < 0.001). Results suggested that the interplay between common garden and genetic cluster for the total number of fruits was due to the *height* and the *number of primary branches*: the Middle Eastern accessions were significantly smaller than the European accessions in Uppsala (Tukey’s HSD = −2.47, *P* < 0.05), and significantly taller in Guangzhou (Tukey’s HSD = 3.53, *P* < 0.01); the Middle Eastern accessions did not have significantly more primary branches than European accessions in Uppsala (Tukey’s HSD = −0.78, *P* = 0.71), while they did in Guangzhou (Tukey’s HSD = 3.33, *P* < 0.01). On the other hand, some patterns were consistent across common gardens: Asian accessions were significantly smaller than European and the Middle Eastern clusters in both common gardens (Tukey’s HSD > 3.70, *P* < 0.01), and likewise, the Middle Eastern cluster had significantly more fruits over a segment of 10 cm than the European cluster in both common gardens (Tukey’s HSD > 3.07, *P* < 0.01). However, Asian accessions were no longer significantly smaller in Guangzhou than the European accessions (*P* = 0.25) after downsampling. Variance of number of fruits was also higher for the Middle Eastern cluster than that of other clusters (Fisher’s *F* > 0.80, *P* < 0.001; **see****Supporting Information—Table S6**), corroborating the hypothesis of higher plasticity of the Middle Eastern cluster, knowing that ME was not more genetically differentiated than the European and Asian clusters (**see****Supporting Information—Fig. S1**; see also Cornille *et al.* 2016).

### Environmental distance was mostly confounded with genetic clusters

When accounting for the effect of both genetic cluster and environmental distance between the sampling site and the common garden (model (2)), statistical analyses suggested a strong genetic cluster effect and an overall weak environmental distance effect: at a Bonferroni adjusted cut-off of 0.0018 (28 comparisons), the genetic cluster effect was significant for 17 out of 28 phenotypes, whereas the environmental distance was significant for none of the 28 phenotypes (though significant for a few phenotypes without the Bonferroni correction; Table 3). In addition, downsampling did not change the trend **[see****Supporting Information****]**. Environmental distance was probably confounded with the effect of genetic clusters, as the genetic cluster effect was significant for 18 out of 28 when excluding environmental distance (model (1), Table 1), and the effect of environmental distance was significant for 3 out of 28 phenotypes when excluding genetic cluster (model (3); **see****Supporting Information—Table S7**). When analysing within a genetic cluster, the effect of environmental distance was negative for most of the traits (e.g. the closer the sampling site is to the common garden, the more performant the accession is), although very few were significant **[see****Supporting Information—Table S8****]**.

**Table 3.** Effects of the environmental distance on each trait of *Capsella bursa-pastoris* in each common garden (Uppsala or Guangzhou), with an analysis of variance of model (2). Statistics for the environmental distance (*χ*_*e*_^2^) and the genetic cluster effect (*χ*_*c*_^2^), and df of the type II Wald chi-square test. GP: time between *sowing* and *germination*; BP: time between *germination* and *bolting*; FP: time between *bolting* and *flowering*; SP: time between *flowering* and *senescence*. The distributions of the residuals are: negative binomial (NB), normal (N) or normal with the starting date as an additional variable (N*). Significant values are bolded. Significance levels are: ***P < 0.001; **P < 0.01; *P < 0.05; ^n.s.^*P* > 0.05.

Trait	Environmental distance and genetic cluster effects								Residual
	Uppsala				Guangzhou				
	*χ*_*e*_^2^	df	*χ*_*c*_^2^	df	*χ*_*e*_^2^	df	*χ*_*c*_^2^	df	
No. of fruits	1.98^n.s.^	1	5.69^n.s.^	2	1.77^n.s.^	1	41.21***	2	NB
Height	0.56^n.s.^	1	70.66***	2	2.64^n.s.^	1	34.54***	2	N
No. of fruits over 10 cm	2.05^**n.s.**^	1	11.21**	2	0.17^n.s.^	1	14.50***	2	N
No. of primary branches	0.27^n.s.^	1	0.43^n.s.^	2	0.13^n.s.^	1	10.37**	2	N
No. of secondary inflorescences	0.95^n.s.^	1	19.75***	2	2.37^n.s.^	1	3.59^n.s.^	2	NB
No. of rosette leaves	4.73*	1	142.56***	2	0.32^n.s.^	1	19.27***	2	N
Rosette diameter	0.78^n.s.^	1	89.30***	2	4.60^n.s.^	1	22.75***	2	N
Germination	0.17^n.s.^	1	42.18***	2	3.49^n.s.^	1	45.62***	2	N
Bolting	0.69^n.s.^	1	22.19***	2	0.56^n.s.^	1	5.68^n.s.^	2	N
BP	3.01^n.s.^	1	92.19***	2	2.62^n.s.^	1	49.31***	2	N*
Flowering	0.03^n.s.^	1	21.61***	2	0.22^n.s.^	1	9.24**	2	N
FP	0.03^n.s.^	1	1.39^n.s.^	2	9.26**	1	29.70***	2	N*
Senescence	6.52*	1	11.35**	2	0.78^n.s.^	1	9.47**	2	N
SP	0.04^n.s.^	1	20.02***	2	0.85^n.s.^	1	7.83*	2	N*

The constraining environment of Uppsala (lower performance on average for most of the traits) compared to Guangzhou probably exacerbated the effect of environmental distance **[see****Supporting Information—Figs. S7**, **S8** and **S9****]**, as it was significant for two phenotypes in Uppsala, but significant for only one in Guangzhou (Table 3). In Uppsala, accessions from distant places had the lowest *number of rosette leaves* (*χ*^2^ = 4.73, df = 1, *P* < 0.05) and the shortest lifespan (*χ*^2^ = 6.52, df = 1, *P* < 0.05).

## Discussion

*Capsella bursa-pastoris* is a tetraploid and self-fertilizing species that expanded worldwide relatively recently (Douglas *et al.* 2015) and populations can today be grouped into at least three main genetic clusters (Cornille *et al.* 2016). We investigated the effect of the genetic clusters in a large-scale experiment with a large number of accessions (223) across two common gardens installed in contrasting environments (Sweden and Eastern China). Our experiment shows that (i) the success of *C. bursa-pastoris* is closely related to its high degree of phenotypic plasticity, (ii) genetic variation had a strong effect on phenotypic variation within a common garden and (iii) the effect of environmental distance was confounded by genetic clusters, impeding a proper assessment of the role of local adaptation.

### The shepherd’s purse is a highly plastic ruderal weed

As stated above, *C. bursa-pastoris* is a species with a worldwide distribution, thus *a priori* facing very contrasted environments, which is expected to favour high phenotypic plasticity for traits associated to fitness (Hendry 2016). In agreement with this expectation and previous studies (Aksoy *et al.* 1998; Caullet 2011; Neuffer 2011; Neuffer *et al.* 2018), our experiment detected phenotypic plasticity for all traits (Fig. 2): all accessions were able to survive in both common gardens, and all showed strong differences in phenotypes between the two common gardens. Though its dissemination is often associated to human activities (Cornille *et al.* 2016), the establishment ability and the worldwide success of *C. bursa-pastoris* were undoubtedly, and at least partly, related to its strong plasticity that is not so common (Palacio-Lopez *et al.* 2015). Phenotypic plasticity of certain morphological traits was related to individual performance (Fig. 3; **see****Supporting Information—Table S5**), where higher plasticity was only beneficial in a richer environment (Guangzhou), but had no effect in poorer environment (Uppsala).

The flexible mating system of *C. bursa-pastoris*, predominantly a selfing species but with an outcrossing rate up to 10 % under field conditions (Hurka *et al.* 1989), probably explains the strong establishment ability. Indeed, controlling the timing of phenology to cope with biotic and abiotic stresses is a response adopted by many plant populations, especially those with an annual life cycle (e.g. Davis *et al.* 2005; Leverett *et al.* 2018). Our experiment clearly showed that phenotypic plasticity of *C. bursa-pastoris* allows a contrasted phenological response, supporting the views of Anderson *et al.* (2012) in *Boechera stricta* (Brassicaeae), or Neuffer and Hurka (1986) that evoked a ‘general purpose genotype’ (a term borrowed from Baker 1965). However, within each common garden, European accessions flowered later than those from other clusters, indicating also a strong genetic determinism. The late flowering causes a longer time span for vegetative growth, and partly explains the significantly larger rosette. An association between flowering trends and rosette characteristics is not surprising. We indeed observed a strong correlation between the rosette size and the flowering time in our study (*r* = 0.52). Linde *et al.* (2001) showed that quantitative trait loci (QTLs) associated with these two traits are closely linked in *C. bursa-pastoris*, and likewise, in *Arabidopsis thaliana*, rosette leaf number has been shown to be sensitive to stimuli of flowering, such as shading (Cookson and Granier 2006) or length of photoperiod (Lewandowska-Sabat *et al.* 2017). The late-flowering phenotype might not be constitutive of all European accessions though, since within small regions and along altitudinal clines in the Alps, highly differing ecotypes from early flowering in the valley to late-flowering types in higher elevations (or even not flowering within one vegetation period) have been recorded (Neuffer and Hurka 1986), which then was again observed along clines in North America (Neuffer and Hurka 1999).

Our study also showed that accessions from the Asian cluster flowered earlier than those from other clusters. Several studies showed that in many species late flowering appears to be the ancestral state (e.g. *A. thaliana*, Le *et al.* 2002; Komeda 2004; *Triticum monococcum*, Yan *et al.* 2003). If it was also the case for *C. bursa-pastoris*, it is probable that the ‘early-flowering’ phenotype recently evolved during *C. bursa-pastoris* range expansion in Asia. Early flowering in Asia can be (i) a response to avoid biotic competition (Orsucci *et al.* 2020), thus compensating the higher genetic load commonly reported in colonization fronts (Excoffier *et al.* 2009); (ii) a response to rather unpredictable environments such as those recorded in Asia (e.g. typhoon, monsoon), as observed in *A. thaliana* (Simpson and Dean 2002; Roux *et al.* 2006); or (iii) a side consequence of a higher sensitivity to photoperiod, as observed in tropical species such as rice (*Oryza sativa*) and maize (*Zea mays*), which is the main cue to the alternation of dry and humid seasons in these climates (Roux *et al.* 2006).

### A strong genetic cluster effect impeding the observation of environmental differentiation

In addition to significant phenotypic plasticity, a clear difference among genetic clusters was observed for almost all traits. We tried to assess whether genetic clustering could be associated with environmental distance, but did not succeed due to confounding between these two factors (see also Samis *et al.* 2019). While our common garden experiment is adequate to study global trend in plasticity and adaptation among genetic clusters, the geographical scale considered here is probably too large to observe local adaptation that may take place at a smaller geographical scale. When investigating within a genetic cluster and at a smaller scale, strong adaptive differentiation can be observed in *A. thaliana* (Ågren and Schemske 2012), a weedy species phylogenetically close to *C. bursa-pastoris* (Beilstein *et al.* 2006). Undoubtedly, local adaptation plays a central role in the establishment success of *C. bursa-pastoris,* but was not detectable at the scale of our study. Alternatively, following the theory that populations shift from phenotypic plasticity at the start of their establishment towards adaptation to local conditions once established (Lande 2015), local adaptation may not have had enough time to be established. An absence of local adaptation coupled with a pronounced plasticity has already been observed in invasive species at the colonization front, such as in *Reynoutria japonica* (VanWallendael *et al.* 2018) or in clonally reproducing macrophytes (*Egeria densa*, *Elodea canadensis* and *Lagarosiphon major*: Riis *et al.* 2010). The absence of local adaptation might also explain the surprisingly low number of genetic clusters given the extensive distribution of *C*. *bursa-pastoris*.

The absence of signature of environmental differentiation might also be explained by pure neutral demographic dynamics, at least for the Asian cluster. Quite consistently, the Asian cluster had a lower performance compared to the other clusters, which might be due to its high genetic load (Cornille *et al.* 2016; Kryvokhyzha *et al.* 2019a, b). The Asian cluster is most certainly a marginal population, and has undergone an accumulation of deleterious mutations, which is common in colonization fronts (Excoffier *et al.* 2009). Although the long-term establishment success can be favoured by high genetic diversity (Hovick and Whitney 2019), a higher genetic load does not necessarily imply a lower performance in the short term (Orsucci *et al.* 2020), and further investigations are needed to account for possible trade-offs between fitness components, especially since germination and seedling stage selection were removed.

## Conclusion

The large-scale of our experiment, involving two common gardens and a large number of accessions representative of the natural range of the shepherd’s purse, allowed us to detect that a relatively high level of phenotypic plasticity is essential for a successful rapid range expansion. For the particular case of *C. bursa-pastoris*, its success appears to be strongly associated to its high phenotypic plasticity and its flexible mating system. Does this indicate that local adaptation does not contribute to a rapid colonization*?* It is probably too early to conclude, but the present study suggests that understanding local adaptation, in *C. bursa-pastoris* or other ruderal species, that went through a rapid range expansion would likely require a combination of more targeted reciprocal transplant experiments as well as experiments at smaller geographical scale.

## Supporting Information

The following additional information is available in the online version of this article—

**Text S1.** Updating the genetic clustering, balancing the block design, the effect of downsampling, part of genetic differentiation vs environmental variation, environmental analyses with bioclimatic variables.

**Table S1.** Average values of temperature, humidity and length of photoperiod at Uppsala and for each month in *Capsella bursa-pastoris*. 

**Table S2.** Average values of temperature, humidity and length of photoperiod at Guangzhou and for each month in *Capsella bursa-pastoris*. 

**Table S3.** Environmental distance from each sampling site to a common garden. 

**Table S4.** Ratio of variance explained by the genetic clusters and the common garden in *Capsella bursa-pastoris*, in terms of sum of squares (model [1]), noted R above. 

**Table S5.** Significance of the link between phenotypic plasticity (*P*-score) and the number of fruits, as described in model (5). 

**Table S6.** Variance ratio of the number of fruits for different genetic clusters (ASI, EUR and ME): statistics and degree of freedom (df) of the Fisher’s *F*-test. 

**Table S7.** Effects of the environmental distance on each trait in *Capsella bursa-pastoris* in each common garden (Uppsala or Guangzhou), with an analysis of variance of model (3). 

**Table S8.** Effect of environmental distance within genetic clusters (ASI, EUR, ME) of *Capsella bursa-pastoris* in each common garden (Uppsala or Guangzhou), with the model (3). 

**Figure S1.** Multi-dimensional scaling of the genotypes (first axis explained 23.47% of the variance, and the second explained 15.74%). 

**Figure S2.** Principal component analysis (PCA) of the environmental data: day length, temperature and humidity. 

**Figure S3.** Partial leverage per sampling site for the number of fruits in Guangzhou in model (2). 

**Figure S4.** Reaction norm of the number of fruits in Uppsala and Guangzhou. 

**Figure S5.** Principal Component Analysis of Figure 2, with PCA 3 and 4, showing the genetic differentiation. 

**Figure S6.** Histograms of the phenological traits of *Capsella bursa-pastoris* (*germination time* in red*, bolting time* in green*, flowering time* in blue, and *senescence time* in purple) according to the common garden and the genetic cluster.

**Figure S7.** Box plots of the *number of fruits* per environmental distance (discretized in intervals), per common gardens, and categorized per genetic cluster: red for ASI, green for EUR, and blue for ME.

**Figure S8.** Table of significance of the model (S1) for each phenotype and for each bioclimatic variable, in Uppsala. 

**Figure S9.** Table of significance of the model (S1) for each phenotype and for each bioclimatic variable, in Guangzhou.

plac011_suppl_Supplementary_Material

## Data Availability

Data and scripts are available from this link: https://forgemia.inra.fr/amandine.cornille/capsella_aobp.

## Notes

**Populations & Communities. Chief Editor**: Jean Burns
