# Supplementary material for: The relative role of plasticity and demographic history in Capsella bursa-pastoris: a common garden experiment in Asia and Europe
Source: AoB Plants. 2022 Apr 2;14(3):plac011. doi: 10.1093/aobpla/plac011 (PMC9162126; doi:10.1093/aobpla/plac011)
Supplement: plac011_suppl_Supplementary_Material [file plac011_suppl_supplementary_material.docx]

## Supplementary materials

### Updating the genetic clustering

Genotyping-by-sequencing (GBS) data for the 267 accessions acquired by Cornille *et al*. (2016, [https://datadryad.org/stash/dataset/doi:10.5061/dryad.71f99](https://datadryad.org/stash/dataset/doi:10.5061/dryad.71f99)) were used to assign accessions to three different clusters (Asia, Middle East or Europe) known to be relevant (Cornille *et al.,* 2016; Kryvokhyzha *et al.*, 2016, 2019a,b). First, we ran a Multi-Dimensional Scaling analysis (function c*mdscale* from the base R package *stats*) on the GBS data that gave the five first principal components on which we ran an unsupervised k-means algorithm with *K = 3* (function *kmeans* from the base R package *stats*). We then tested its robustness by using a random forest approach (function *randomForest* from the R package *randomForest*; Liaw & Wiener, 2002), with a bootstrap (1,000 iterations) of 20% of the dataset (80% being used as a training set). Only accessions that were assigned more than 95% of the iterations to the same cluster were kept for further analyses, reducing the dataset from 267 accessions to 232 accessions.

### Balancing the block design

- - 1. Before each statistical analysis, the block design was artificially balanced by down sampling the dataset down to four replicates per accession: we kept the best combination of four blocks (out of six) in terms of the number of accessions present in all the four blocks. This filtering was applied for each trait, not necessarily ending up with the same sample each time.

### The effect of downsampling

- - 1. Since the number of accessions from the different clusters was variable we have re-analysed the data by downsampling the dataset to have a balanced number of accessions from each genetic clusters Asian, European and Middle-Eastern). After balancing for the block design, as the Middle-Eastern cluster was constituted of 27 accessions, we randomly sampled 27 accessions from the Asian cluster and 27 accessions from the European cluster. We performed this random downsampling 100 times per trait and per common garden, resulting in a distribution of *χ^2^* statistics for the analysis of variance. We then averaged the *χ^2^* statistics per trait and per common garden, and computed the corresponding p-values.
    2. In terms of significance of the *χ^2^* statistics, 19 values out of 269 values changed: in Table 1 (out of 28 values), the genetic cluster for the total number of fruits was no longer significant in Uppsala (*p =* 0.12). In Table 2 (out of 84 values), the difference between EUR and ASI was no longer significant for the total number of fruits (*p =* 0.38), BT (*p =* 0.13), and number of secondary inflorescence (*p =* 0.17) in Uppsala, and for total number of fruits (*p =* 0.13), height (*p =* 0.25), number of fruits over 10 cm (*p =* 0.15), BT (*p =* 0.18), FP (*p =* 0.13), and SP (*p =* 0.18) in Guangzhou. The difference between EUR and ME was no longer significant for height (*p =* 0.12) in Uppsala, and for FT (*p =* 0.07) and ST (*p =* 0.06) in Guangzhou. In Table 3 (out of 56 values), the environmental effect became significant in Guangzhou for BT (*p* < 0.05). In Table S4 (out of 14 values): the sign of the ratio did not change for any values. In Table S5 (out of 56 values): no significance changed. In Table S6 (out of 3 values): no significance changed. In Table S7 (out of 28 values): the environmental distance effect was no longer significant in Uppsala for the rosette diameter (*p =* 0.20), BT (*p =* 0.21), and BP (*p =* 0.31), and in Guangzhou for the rosette diameter (*p =* 0.24), FT (*p =* 0.18), and SP (*p =* 0.38).
    3. Please note that the higher phenotypic variance found in European accessions is greatly due to the larger sampling range compared to the other two clusters. Therefore, downsampling mostly affected the European cluster and reflected the location of the accessions rather than the effect of the uneven sampling size. However, this analysis shows that our results are generally robust to uneven sampling sizes as only 19 out of the 269 p-values changed.

### Part of genetic differentiation vs environmental variation

In order to quantify the relative importance of phenotypic variation due to environment compared to genetic differentiation, we considered the ratio *R_i_* for trait *i* of the sum of squares of the genetic cluster effect (*c_i_*) and the site effect (*s_j_*) in model [1]. If *R_i_* is higher than 1, the contribution of genetic differentiation can be considered to be dominant compared to that of phenotypic plasticity. The result is summarized in Table S3.

### Environmental analyses with bioclimatic variables

We pushed the analyses of the environmental distance (model [2]) further by using directly the bioclimatic variables in the statistical models. We then fitted the following model:

*Y_ijkl_ ~ μ + a_k_ + c_i_ + B_j_ + A_k_ + e_ijkl_* [S1]

where “a*_k_”* is one of the 19 bioclimatic variables (or latitude or longitude), and the rest is as described for model [2] (the residual distributions are summarized in Table 1). As for model [2], we removed the north-eastern Chinese accessions from the analysis.

As a result, a high number of bioclimatic variables showed a significant association to the traits, in both common gardens (Fig. S8 and S9). Most of the significant associations were not common among common gardens. As for the environmental distance in model [2], the number of significant associations were larger in Uppsala (101 out of 294) than in Guangzhou (33 out of 294). With a Bonferroni adjusted cut-off of 0.00017 (294 comparisons), only seven was significant in Uppsala, and none in Guangzhou.

Every phenotype with several significant bioclimatic variables were also significantly associated with the environmental distance in model [2] (Table 2, Fig. S8 and S9), supporting the analysis of model [2]. However, the converse was not always true, as for the *number of primary branches* and the *number of secondary inflorescences*.

In Uppsala, most of the phenology was significantly associated with bioclimatic variables, slightly biased towards the temperatures of home environment (bio1 and bio3 to bio11 in Fig. S8 and S9). The *number of rosette leaves* and the *number of secondary inflorescences* were significantly associated to most of the bioclimatic variables. The *number of primary branches* and the *number of fruits* were significantly associated with temperatures. In Guangzhou, only *FP* was consistently associated with precipitation bioclimatic variables (bio12 to bio19); *BP, the number of primary branches* and *height* were mostly associated to temperature bioclimatic variables.

Even without the north-eastern Chinese accessions, almost every analysis of the model (S1) showed some points with a high partial leverage value (data not shown): these analyses should be taken with caution. The main difference between the analysis of environmental distance and that of the bioclimatic variables is probably due to the PCA sorting out the inconsistent variance.

## References

**Cornille A, Salcedo A, Kryvokhyzha D, Glémin S, Holm K, Wright S et al. 2016.** Genomic signature of successful colonization of Eurasia by the allopolyploid shepherd's purse (*Capsella bursa-pastoris*). *Molecular ecology* 25: 616-629.

**Kryvokhyzha D, Holm K, Chen J, Cornille A, Glémin S, Wright SI. et al. 2016.** The influence of population structure on gene expression and flowering time variation in the ubiquitous weed *Capsella bursa-pastoris* (Brassicaceae). *Molecular Ecology* **25**: 1106-1121.

**Kryvokhyzha D, Milesi P, Duan T, Orsucci M, Wright SI, Glémin S. et al.** **2019a.** Towards the new normal: Transcriptomic convergence and genomic legacy of the two subgenomes of an allopolyploid weed (*Capsella bursa-pastoris*). *PLoS genetics* **15**: e1008131.

**Kryvokhyzha D, Salcedo A, Eriksson MC, Duan T, Tawari N, Chen J. et al. 2019b.** Parental legacy, demography, and admixture influenced the evolution of the two subgenomes of the tetraploid *Capsella bursa-pastoris* (Brassicaceae). *PLoS genetics* **15**: e1007949.

**Liaw A, Wiener M. 2002.** Classification and regression by randomForest. *R news* **2**: 18-22.

## Tables

1. **Table S1.** Average values of temperature, humidity and length of photoperiod at Uppsala and for each month in *Capsella bursa-pastoris*. First and last dates were indicated in the first and last months of the experiment.

| **Uppsala** | **2014** | | | | |
| --- | --- | --- | --- | --- | --- |
|  | **May (> 1st)** | **Jun.** | **Jul.** | **Aug.** | **Sep. (< 13th)** |
| **Temperature (°C)** | 10.7 | 13.4 | 19.5 | 17.7 | 14.2 |
| **Humidity (%)** | 72.0 | 73.9 | 73.9 | 82.2 | 86.3 |
| **Photoperiod (h)** | 16.9 | 18.6 | 18.1 | 15.8 | 13.7 |

**Table S2.** Average values of temperature, humidity and length of photoperiod at Guangzhou and for each month in *Capsella bursa-pastoris*. First and last dates were indicated in the first and last months of the experiment.

| **Guangzhou** | **2014** | | **2015** | | | | |
| --- | --- | --- | --- | --- | --- | --- | --- |
|  | **Nov. (> 1st)** | **Dec.** | **Jan.** | **Feb.** | **Mar.** | **Apr.** | **May (< 12th)** |
| **Temperature (°C)** | 20.4 | 14.4 | 14.6 | 17.8 | 21.7 | 23.5 | 26.8 |
| **Humidity (%)** | 76.6 | 74.6 | 78.1 | 84.2 | 88.5 | 82.6 | 86.3 |
| **Photoperiod (h)** | 10.7 | 10.9 | 11.4 | 12.0 | 12.7 | 13.2 | 13.5 |

**Table S3.** Environmental distance from each sampling site to a common garden. The distances were averaged over the 234 accessions.

| **Region** | **Country** | **Cluster** | **Distance to Guangzhou** | **Distance to Uppsala** |
| --- | --- | --- | --- | --- |
| Asia | China | ASI | 5.07 | 5.62 |
| Asia | China | EUR | 9.63 | 6.33 |
| Europe | Czech Republic | EUR | 7.55 | 1.70 |
| Europe | France | EUR | 7.18 | 4.74 |
| Europe | Italy | ME | 6.71 | 5.16 |
| Europe | Russia | EUR | 10.3 | 5.64 |
| Europe | Spain | EUR | 7.44 | 3.29 |
| Europe | Sweden | EUR | 8.43 | 1.15 |
| Europe | United Kingdom | EUR | 8.26 | 3.29 |
| Middle-East | Algeria | ME | 7.11 | 5.39 |
| Middle-East | Israel | ME | 7.02 | 6.99 |
| Middle-East | Jordan | ME | 8.28 | 6.26 |
| Middle-East | Syria | ME | 8.65 | 6.84 |
| Middle-East | Turkey | ME | 6.61 | 3.92 |

**Table S4.** Ratio of variance explained by the genetic clusters and the common garden in *Capsella bursa-pastoris*, in terms of sum of squares (model [1]), noted R above. GP: time between *sowing* and *germination*; BP: time between *germination* and *bolting*; FP: time between *bolting* and *flowering*; SP: time between *flowering* and *senescence*. The distributions of the residual are: negative binomial (NB) Normal (N) or Normal with the starting date as an additional variable (N*).

| **Traits** | **Genetic differentiation over phenotypic plasticity** | **Residual** |
| --- | --- | --- |
| **Nb. of fruits** | 0.15 | NB |
| **Height** | 0.49 | N |
| **Nb. of fruits over 10 cm** | 1.69 | N |
| **Nb. of primary branches** | 0.03 | N |
| **Nb. of secondary inflorescences** | 0.22 | NB |
| **Nb. of rosette leaves** | 43.5 | N |
| **Rosette diameter** | 0.17 | N |
| **Germination** | 0.35 | N |
| **Bolting** | 0.003 | N |
| **BP** | 0.90 | N* |
| **Flowering** | 0.002 | N |
| **FP** | 40.7 | N* |
| **Senescence** | 0.01 | N |
| **SP** | 0.08 | N* |

**Table S5.** Significance of the link between phenotypic plasticity (*P*-score) and the number of fruits, as described in model [5]. The Fisher’s *F* statistics, degrees of freedom and the p-values are reported. Significance levels are: *p**** < 0.001; *p** <* 0.01; *p** < 0.05; *p^n.s.^* > 0.05.

| **Traits** | **Global** | | | **ASI** | | | **EUR** | | | **ME** | | |
| --- | --- | --- | --- | --- | --- | --- | --- | --- | --- | --- | --- | --- |
|  | **F** | **df1** | **df2** | **F** | **df1** | **df2** | **F** | **df1** | **df2** | **F** | **df1** | **df2** |
| Height | **45.4***** | 1 | 67 | **19.3***** | 1 | 31 | 0.1^n.s.^ | 1 | 14 | **20.2***** | 1 | 18 |
| Nb. of fruits over 10 cm | **17.8***** | 1 | 67 | **8.2**** | 1 | 31 | **6.9*** | 1 | 14 | 2.1^n.s.^ | 1 | 18 |
| Nb. of primary branches | **32.4***** | 1 | 67 | **8.1**** | 1 | 31 | **9.7**** | 1 | 14 | 2.4^n.s.^ | 1 | 18 |
| Nb. of secondary inflorescences | 0.0005^n.s.^ | 1 | 67 | 0.3^n.s.^ | 1 | 31 | 0.4^n.s.^ | 1 | 14 | 1.9 x 10^-6 n.s.^ | 1 | 18 |
| Nb. of rosette leaves | 0.005^n.s.^ | 1 | 44 | **4.7*** | 1 | 21 | 0.8^n.s.^ | 1 | 2 | **4.6*** | 1 | 17 |
| Rosette diameter | **5.7*** | 1 | 43 | 0.8^n.s.^ | 1 | 20 | 0.4^n.s.^ | 1 | 2 | 0.4^n.s.^ | 1 | 17 |
| Germination | 0.08^n.s.^ | 1 | 67 | 1.3^n.s.^ | 1 | 31 | **5.5*** | 1 | 14 | 0.1^n.s.^ | 1 | 18 |
| Bolting | **4.0*** | 1 | 58 | 2.0^n.s.^ | 1 | 27 | 1.3^n.s.^ | 1 | 12 | 0.2^n.s.^ | 1 | 15 |
| BP | **4.5*** | 1 | 58 | 2.2^n.s.^ | 1 | 27 | 1.0^n.s.^ | 1 | 12 | 0.05^n.s.^ | 1 | 15 |
| Flowering | 1.6^n.s.^ | 1 | 44 | 2.3^n.s.^ | 1 | 21 | 0.2^n.s.^ | 1 | 2 | 2.3^n.s.^ | 1 | 17 |
| FP | **4.6*** | 1 | 39 | 0.3^n.s.^ | 1 | 19 | 0.5^n.s.^ | 1 | 2 | 1.7^n.s.^ | 1 | 14 |
| Senescence | 0.2^n.s.^ | 1 | 60 | 0.3^n.s.^ | 1 | 29 | 2.0^n.s.^ | 1 | 12 | 0.02^n.s.^ | 1 | 15 |
| SP | 0.04^n.s.^ | 1 | 41 | 1.5^n.s.^ | 1 | 20 | 2.2^n.s.^ | 1 | 3 | **7.5*** | 1 | 14 |

**Table S6.** Variance ratio of the number of fruits for different genetic clusters (ASI, EUR and ME): statistics and degree of freedom (df) of the Fisher’s *F*-test. Significance levels are: *p**** < 0.001; *p** <* 0.01; *p** < 0.05; *p^n.s.^* > 0.05.

| **Hypothesis** | **Estimate** | **df (numerator/denominator)** |
| --- | --- | --- |
| ASI/EUR = 1 | 0.80*** | 1422/905 |
| ME/ASI = 1 | 2.23*** | 461/1422 |
| ME/EUR = 1 | 1.79*** | 461/905 |

**Table S7.** Effects of the environmental distance on each trait in *Capsella bursa-pastoris* in each common garden (Uppsala or Guangzhou), with an analysis of variance of model [3]. Statistics for *envd* (*χ_e_^2^*), and degree of freedom (df) of the type II Wald chi-square test. GP: time between *sowing* and *germination*; BP: time between *germination* and *bolting*; FP: time between *bolting* and *flowering*; SP: time between *flowering* and *senescence*. The distributions of the residual are: negative binomial (NB) Normal (N) or Normal with the starting date as an additional variable (N*). Significance levels are: *p**** < 0.001; *p** <* 0.01; *p** < 0.05; *p^n.s.^* > 0.05.

| Trait | **Environmental distance effect** | | | | Residual |
| --- | --- | --- | --- | --- | --- |
|  | **Uppsala** | | **Guangzhou** | |  |
|  | 1. ***χ_e_^2^*** | **df** | 1. ***χ_e_^2^*** | **df** |  |
| Nb. of fruits | 3.35*^n.s.^* | 1 | 1.02*^n.s.^* | 1 | NB |
| Height | **5.81*** | 1 | 3.66*^n.s.^* | 1 | N |
| Nb. of fruits over 10 cm | 2.76*^n.s.^* | 1 | 0.86*^n.s.^* | 1 | N |
| Nb. of primary branches | 0.47*^n.s.^* | 1 | 0.72*^n.s.^* | 1 | N |
| Nb. of secondary inflorescences | 0.02*^n.s.^* | 1 | 0.0008*^n.s.^* | 1 | NB |
| Nb. of rosette leaves | 1.23*^n.s.^* | 1 | **22.3***** | 1 | N |
| Rosette diameter | **6.78**** | 1 | **5.76*** | 1 | N |
| Germination | 1.34*^n.s.^* | 1 | **77.8***** | 1 | N |
| Bolting | **4.52*** | 1 | 3.69*^n.s.^* | 1 | N |
| BP | **9.42**** | 1 | **76.9***** | 1 | N* |
| Flowering | 1.34*^n.s.^* | 1 | **8.39**** | 1 | N |
| FP | 0.002*^n.s.^* | 1 | 0.91*^n.s.^* | 1 | N* |
| Senescence | **5.34*** | 1 | 0.02*^n.s.^* | 1 | N |
| SP | 1.02*^n.s.^* | 1 | **4.12*** | 1 | N* |

**Table S8.** Effect of environmental distance within genetic clusters (ASI, EUR, ME) of *Capsella bursa-pastoris* in each common garden (Uppsala or Guangzhou), with the model [3]. GP: time between *sowing* and *germination*; BP: time between *germination* and *bolting*; FP: time between *bolting* and *flowering*; SP: time between *flowering* and *senescence*. The distributions of the residual are: negative binomial (NB) Normal (N) or Normal with the starting date as an additional variable (N*). Significance levels are: *p**** < 0.001; *p** <* 0.01; *p** < 0.05; *p^n.s.^* > 0.05 (type II Wald chi-square test, with degree of freedom equal to 1).

| **Trait** | **Environmental distance** | | | | | | **Residual** |
| --- | --- | --- | --- | --- | --- | --- | --- |
|  | **Uppsala** | | | **Guangzhou** | | |  |
|  | **ASI** | **EUR** | **ME** | **ASI** | **EUR** | **ME** |  |
| **Nb. of fruits** | -0.07*^n.s.^* | -0.04*^n.s.^* | -0.06*^n.s.^* | -0.05*^n.s.^* | -0.06*^n.s.^* | 0.05*^n.s.^* | NB |
| **Height** | 7.28*^n.s.^* | 1.44*^n.s.^* | **-21.13*** | -11.57*^n.s.^* | -18.22*^n.s.^* | 3.38*^n.s.^* | N |
| **Nb. of fruits over 10 cm** | -1.70*^n.s.^* | -0.44*^n.s.^* | 0.39*^n.s.^* | -0.34*^n.s.^* | -0.04*^n.s.^* | 1.03*^n.s.^* | N |
| **Nb. of primary branches** | -0.15*^n.s.^* | 0.01*^n.s.^* | 0.04*^n.s.^* | -0.18*^n.s.^* | 0.05*^n.s.^* | 0.57*^n.s.^* | N |
| **Nb. of secondary inflorescences** | **0.21*** | -0.03*^n.s.^* | 0.02*^n.s.^* | -0.03*^n.s.^* | -0.05*^n.s.^* | -0.06*^n.s.^* | NB |
| **Nb. of rosette leaves** | -1.26*^n.s.^* | **2.48**** | -0.96*^n.s.^* | 0.65*^n.s.^* | -1.16*^n.s.^* | -0.79*^n.s.^* | N |
| **Rosette diameter** | -5.40*^n.s.^* | -0.01*^n.s.^* | -2.99*^n.s.^* | -5.21*^n.s.^* | **-12.10*** | -11.96*^n.s.^* | N |
| **Germination** | 0.13*^n.s.^* | 0.07*^n.s.^* | 0.09*^n.s.^* | -0.12*^n.s.^* | -0.07*^n.s.^* | -0.09*^n.s.^* | N |
| **Bolting** | **-1.35*** | 0.19*^n.s.^* | -0.77*^n.s.^* | **-2.32*** | **-2.02*** | **-3.02*** | N |
| **BP** | **-0.51*** | -0.03*^n.s.^* | -0.07*^n.s.^* | 0.19*^n.s.^* | 0.05*^n.s.^* | 0.13*^n.s.^* | N* |
| **Flowering** | -0.95*^n.s.^* | 0.62*^n.s.^* | -0.41*^n.s.^* | -1.81*^n.s.^* | -1.42*^n.s.^* | **-3.43*** | N |
| **FP** | 0.09*^n.s.^* | -0.09*^n.s.^* | **0.95**** | **-0.42**** | -0.15*^n.s.^* | 0.31*^n.s.^* | N* |
| **Senescence** | -0.24*^n.s.^* | **-0.62**** | -0.04*^n.s.^* | 0.44*^n.s.^* | -0.58*^n.s.^* | -1.84*^n.s.^* | N |
| **SP** | 1.00*^n.s.^* | -0.69*^n.s.^* | 0.50*^n.s.^* | -1.38*^n.s.^* | **2.08*** | **3.22*** | N* |

## Figures


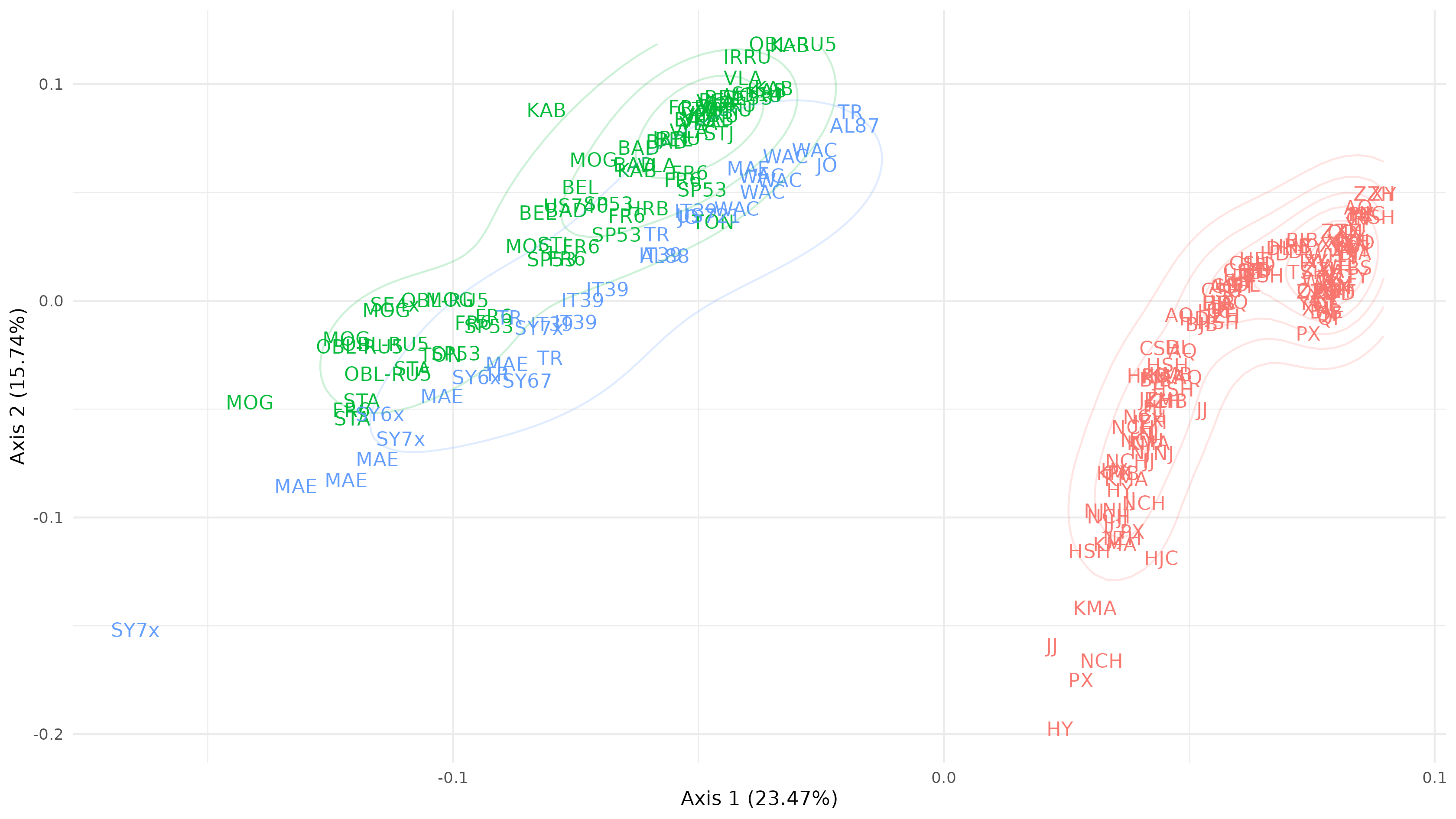


**Figure S1.** Multi Dimensional Scaling of the genotypes(first axis explained 23.47% of the variance, and the second explained 15.74%). Each label is a sampling site, and each colour is a genetic cluster (EUR: green; ASI: red; and ME: blue).


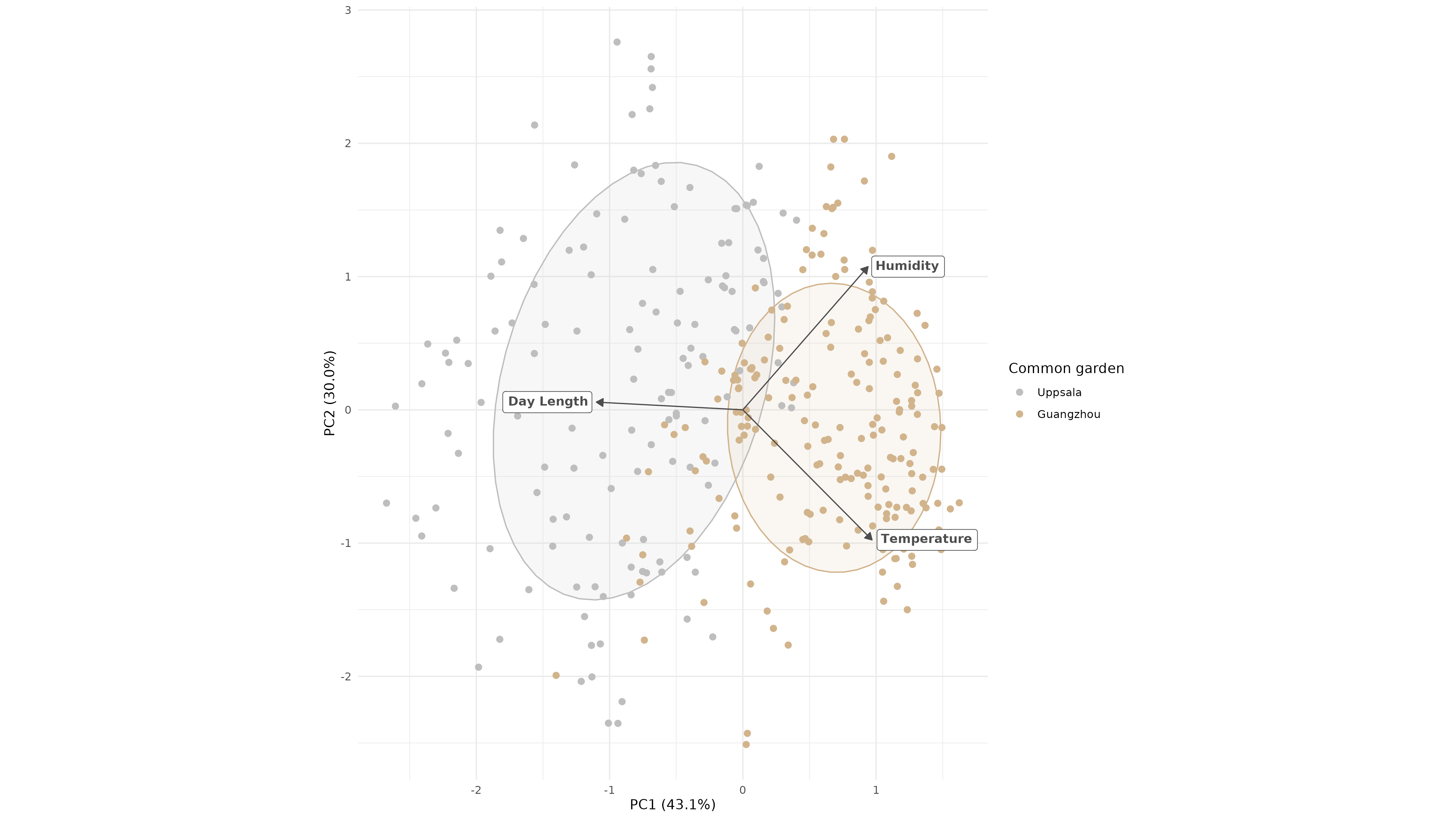


**Figure S2.** Principal component analysis (PCA) of the environmental data: day length, temperature and humidity. Dots correspond to daily records: 139 records for Uppsala (gray) and 193 for Guangzhou (beige). PC1 captured 43.1% of the variance, and PC2 captured 30.0%. Ellipses correspond to 60% confidence ellipses.


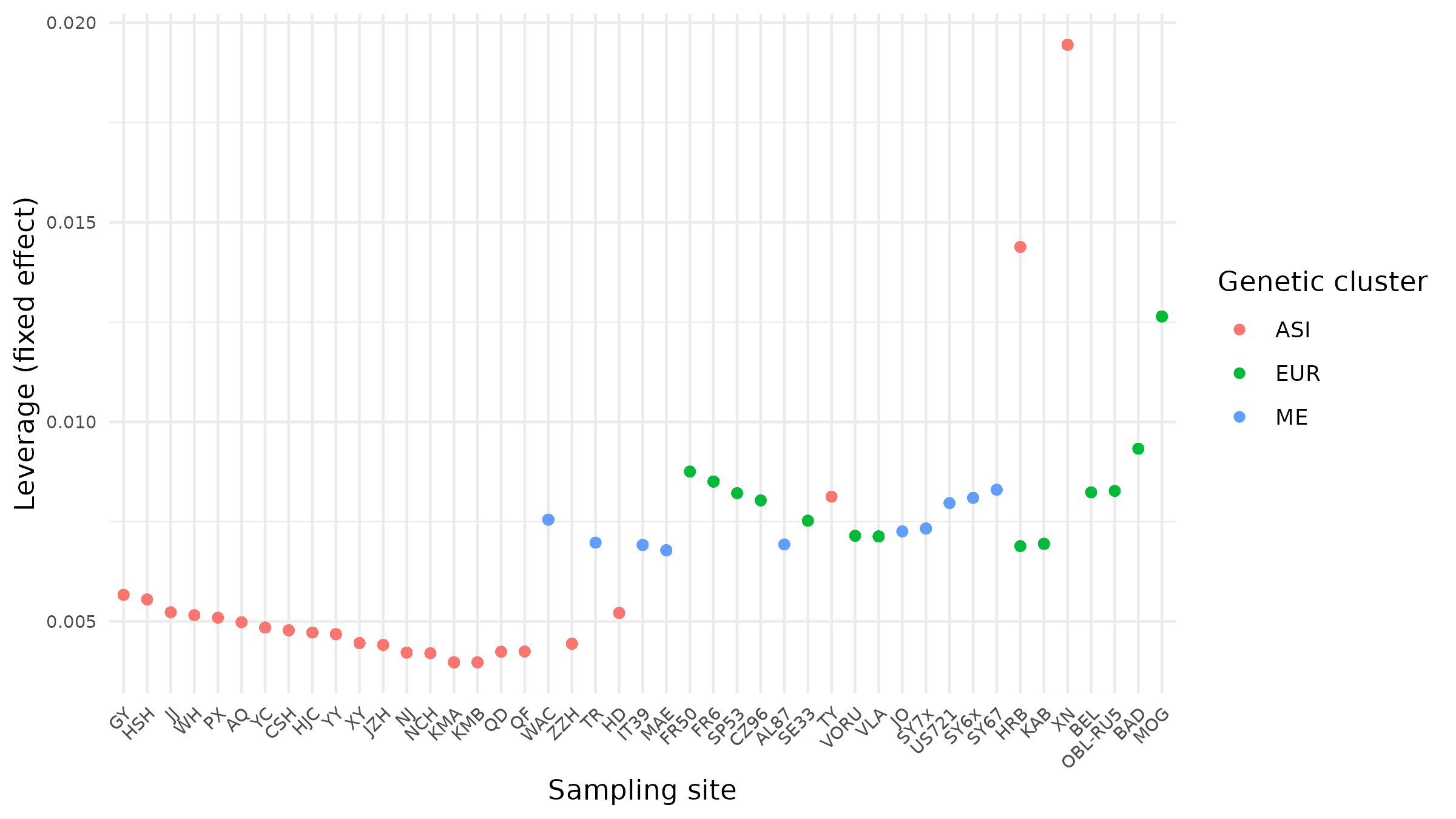
**Figure S3.** Partial leverage per sampling site for the number of fruits in Guangzhou in model [2]. On the x-axis, sampling site are ordered from the closest to the farthest point to Guangzhou in terms of environmental distance. The rightmost sampling sites (HRB, KAB, XN, BEL, OBL-RU5, BAD, MOG) are from the eastern Russia or the north-eastern China (for more details on the sampling site, see Cornille *et al*., 2016; Kryvokhyzha *et al*., 2016; Kryvokhyzha *et al*. 2019b).


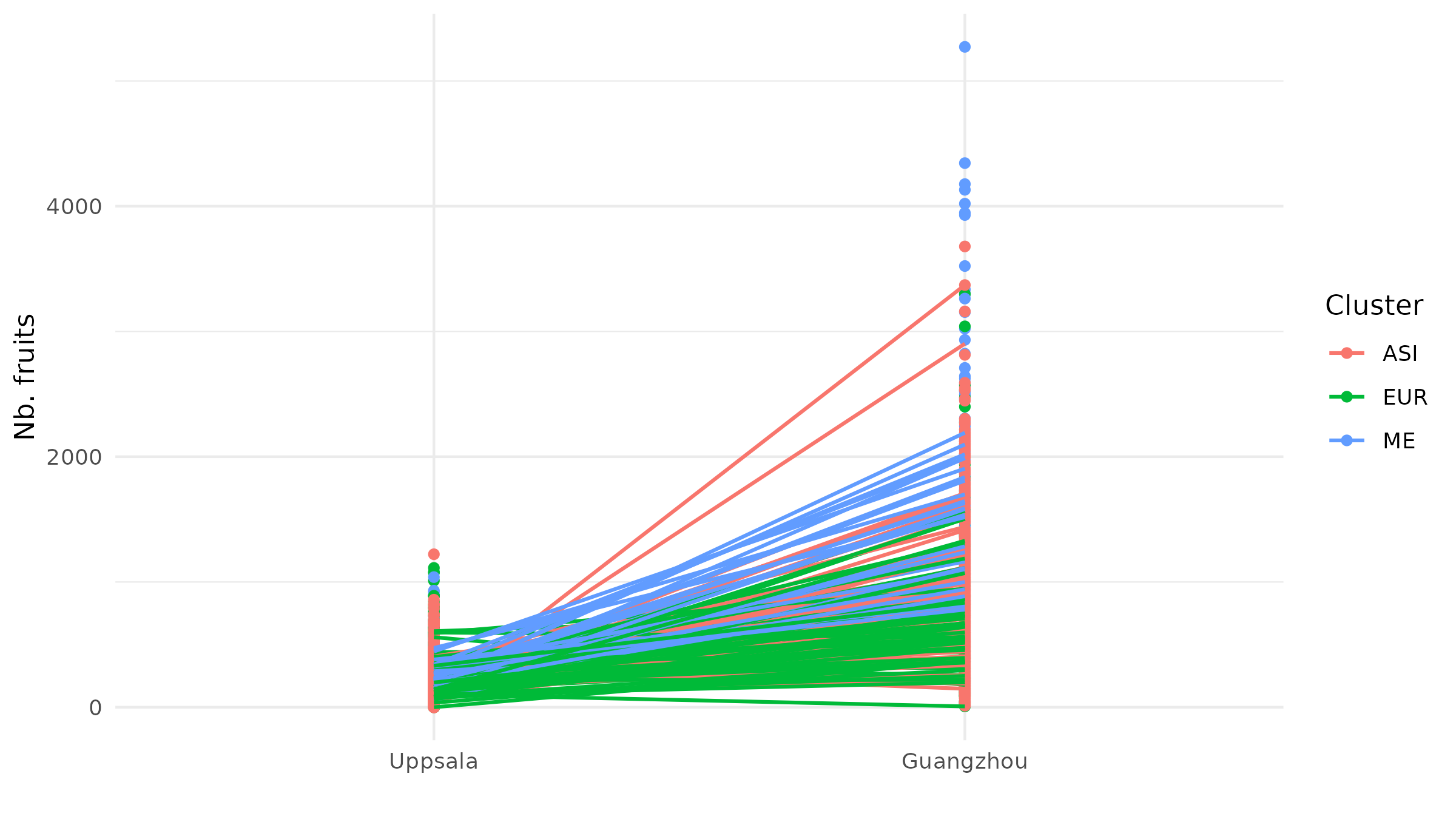
**Figure S4.** Reaction norm of the number of fruits in Uppsala and Guangzhou. Each point is an accession. Each line is a regression for a given accession, and is categorized per genetic cluster: red for ASI, green for EUR, and blue for ME.


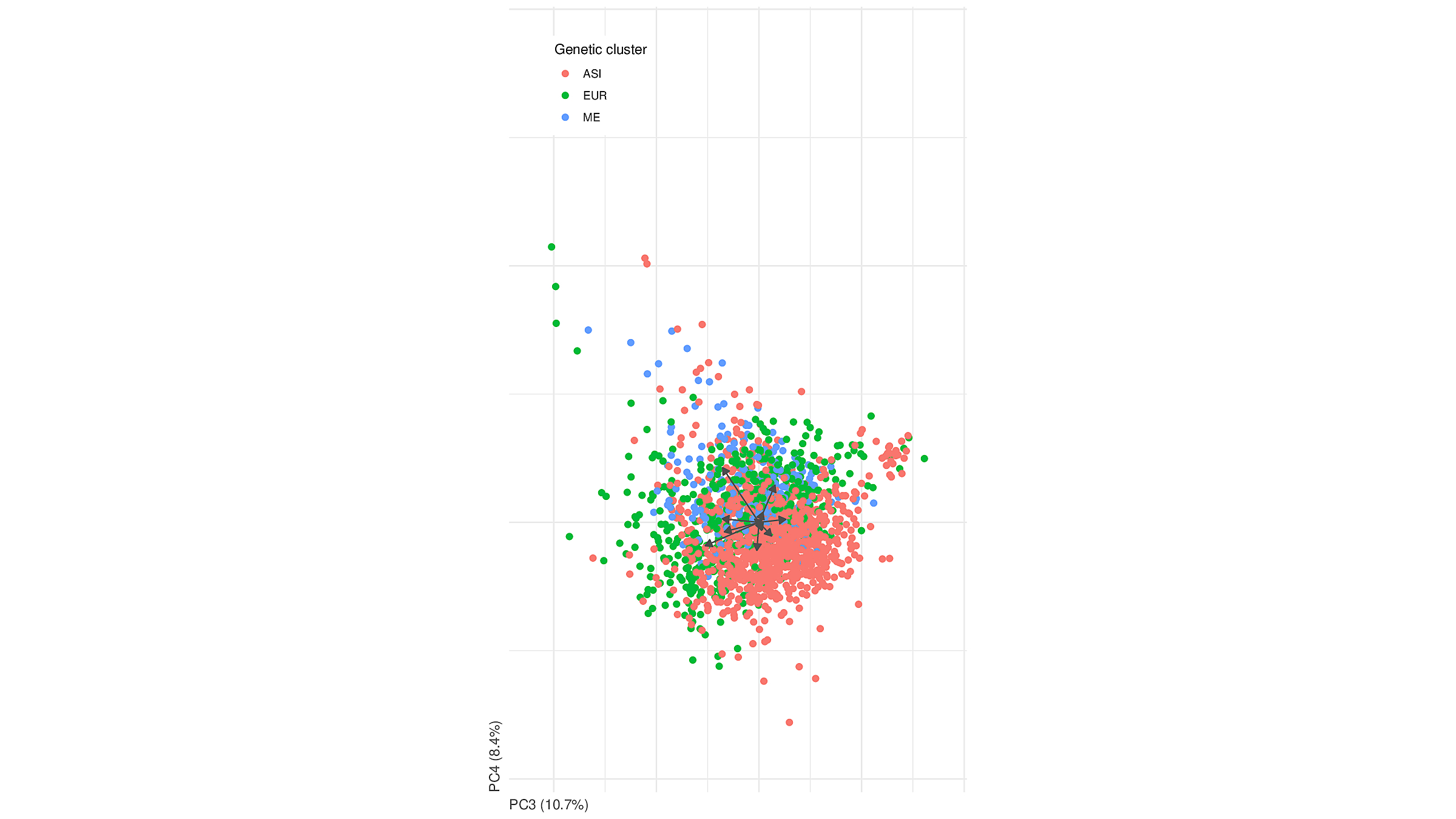


**Figure S5.** Principal Component Analysis of Figure 2, with PCA 3 and 4, showing the genetic differentiation.


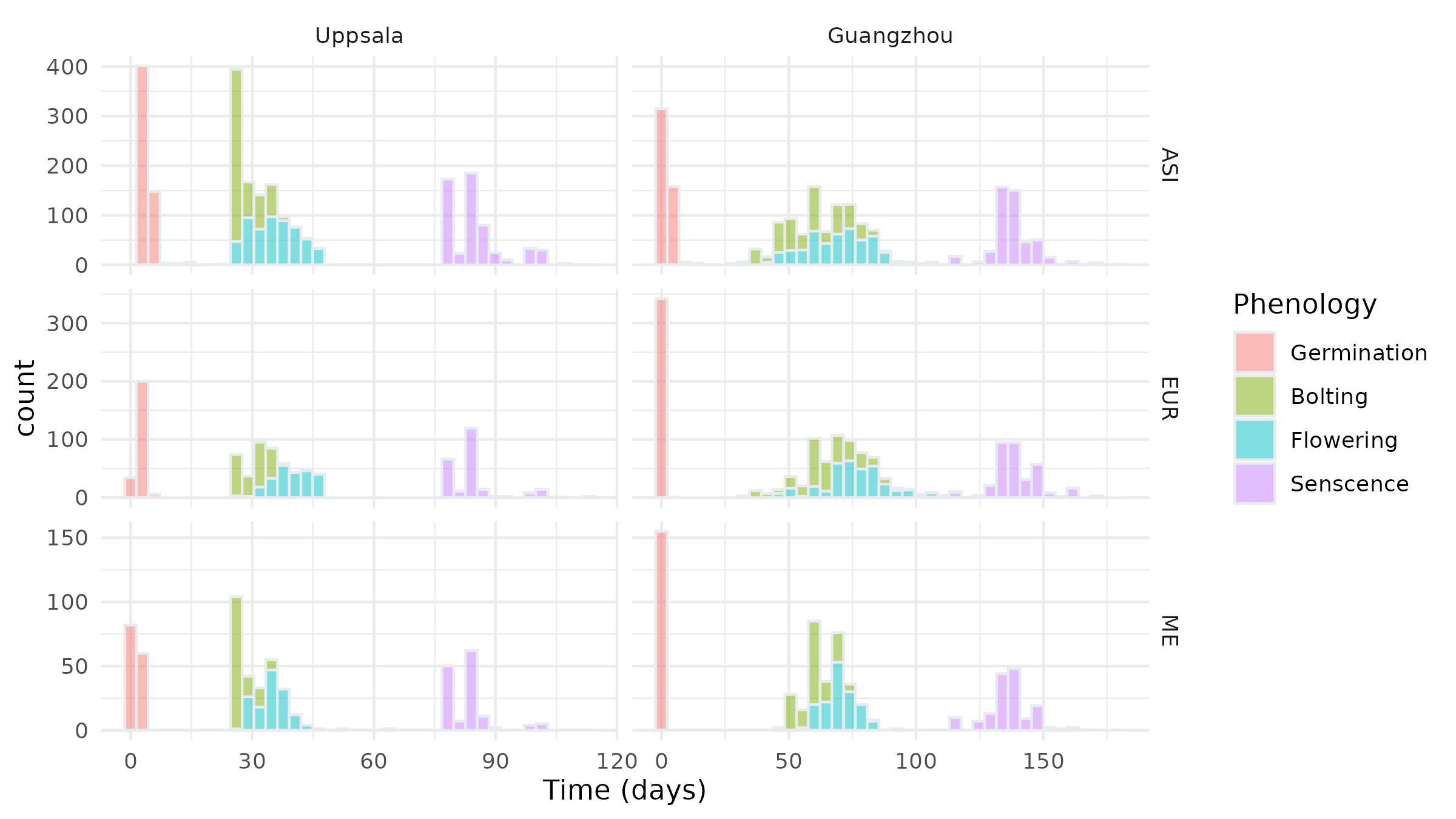
**Figure S6.** Histograms of the phenological traits of *Capsella bursa-pastoris* (*germination time* in red*, bolting time* in green*, flowering time* in blue*,* and *senescence time* in purple) according to the common garden and the genetic cluster.


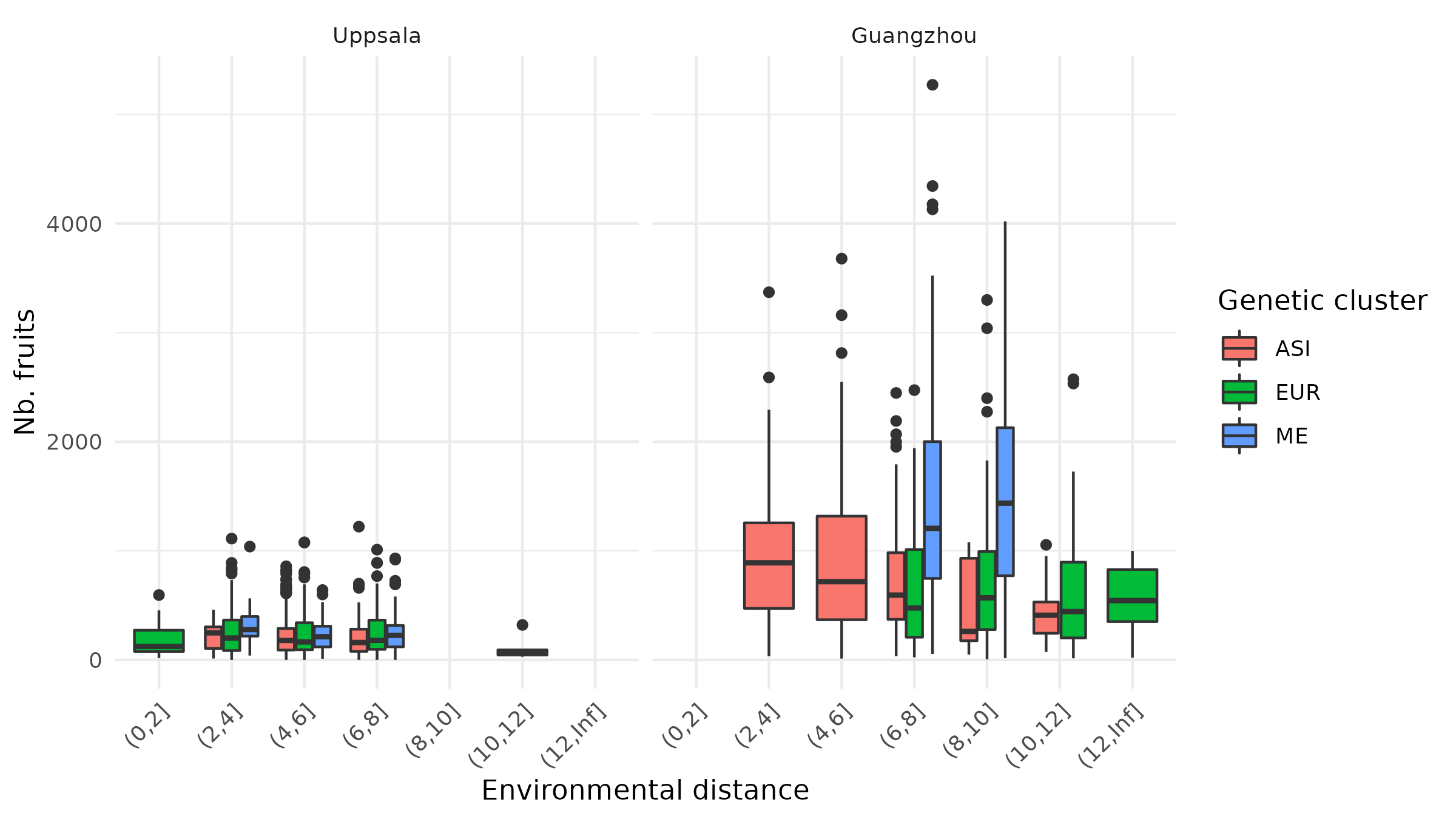
**Figure S7.** Box plots of the *number of fruits* per environmental distance (discretized in intervals), per common gardens, and categorized per genetic cluster: red for ASI, green for EUR, and blue for ME.


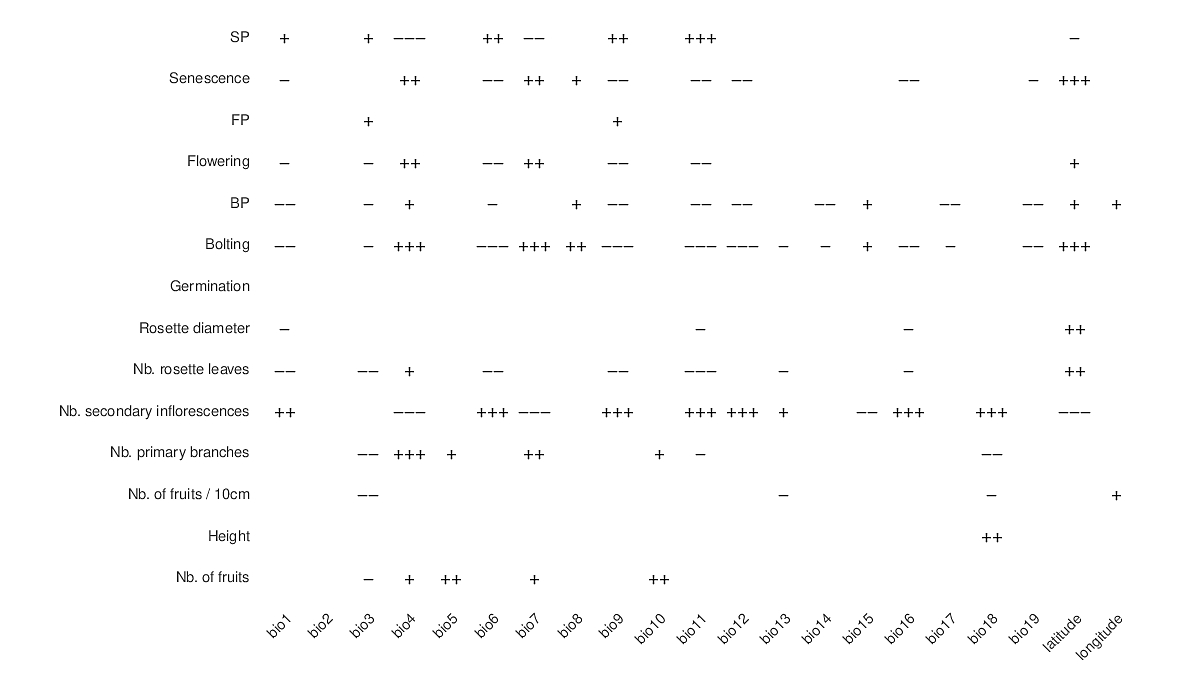


**Figure S8.** Table of significance of the model (S1) for each phenotype and for each bioclimatic variable, in Uppsala. The symbols (‘+’ or ‘-’) indicates the sign of the estimated effect of the bioclimatic variable, and the number of the symbols indicates its significance: +++: p < 0.001; ++: p < 0.01; +: p < 0.05.


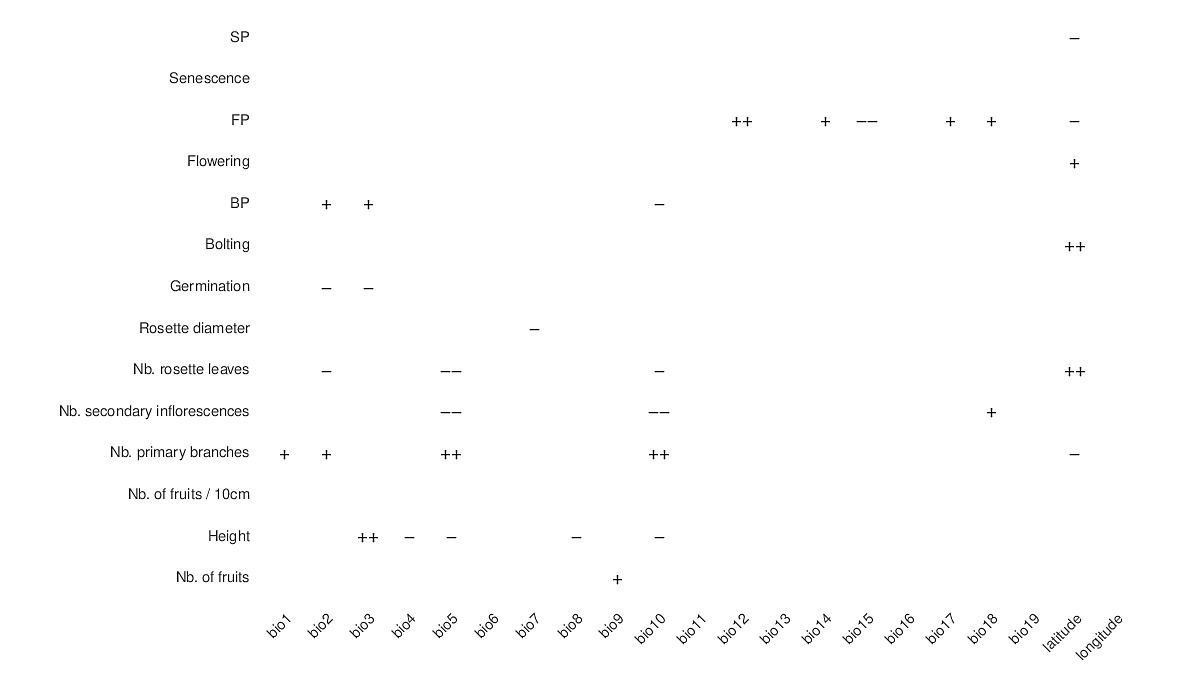
**Figure S9.** Table of significance of the model (S1) for each phenotype and for each bioclimatic variable, in Guangzhou. The symbols (‘+’ or ‘-’) indicates the sign of the estimated effect of the bioclimatic variable, and the number of the symbols indicates its significance: +++: p < 0.001; ++: p < 0.01; +: p < 0.05.
